# HSP90 inhibitor NVP-BEP800 affects stability of SRC kinases and growth of T-cell and B-cell acute lymphoblastic leukemias

**DOI:** 10.1038/s41408-021-00450-2

**Published:** 2021-03-18

**Authors:** Rony Mshaik, John Simonet, Aleksandra Georgievski, Layla Jamal, Shaliha Bechoua, Paola Ballerini, Pierre-Simon Bellaye, Zandile Mlamla, Jean-Paul Pais de Barros, Audrey Geissler, Pierre-Jean Francin, François Girodon, Carmen Garrido, Ronan Quéré

**Affiliations:** 1 UMR1231, Inserm, Université de Bourgogne Franche-Comté, Dijon, France; 2 LipSTIC LabEx, Fondation de Coopération Scientifique de Bourgogne Franche-Comté, Dijon, France; 3 Centre de Ressources Biologiques Ferdinand Cabanne, Hôpital Universitaire François Mitterrand, Dijon, France; 4 Laboratoire d’Hématologie, Assistance Publique Hôpitaux de Paris, Hôpital Armand Trousseau, Paris, France; 5 Centre Georges-François Leclerc, Dijon, France; 6 Plateforme de Lipidomique, Université de Bourgogne Franche-Comté, Dijon, France; 7 Plateforme d’Imagerie Cellulaire, CellImaP, Université de Bourgogne Franche-Comté, Dijon, France; 8 Laboratoire de Génétique Chromosomique et Moléculaire, Plateau Technique de Biologie, Hôpital Universitaire François Mitterrand, Dijon, France; 9 Service d’Hématologie Biologique, Hôpital Universitaire François Mitterrand, Dijon, France

**Keywords:** Acute lymphocytic leukaemia, Acute lymphocytic leukaemia

## Abstract

T-cell and B-cell acute lymphoblastic leukemias (T-ALL, B-ALL) are aggressive hematological malignancies characterized by an accumulation of immature T- or B-cells. Although patient outcomes have improved, novel targeted therapies are needed to reduce the intensity of chemotherapy and improve the prognosis of high-risk patients. Using cell lines, primary cells and patient-derived xenograft (PDX) models, we demonstrate that ALL cells viability is sensitive to NVP-BEP800, an ATP-competitive inhibitor of Heat shock protein 90 (HSP90). Furthermore, we reveal that lymphocyte-specific SRC family kinases (SFK) are important clients of the HSP90 chaperone in ALL. When PDX mice are treated with NVP-BEP800, we found that there is a decrease in ALL progression. Together, these results demonstrate that the chaperoning of SFK by HSP90 is involved in the growth of ALL. These novel findings provide an alternative approach to target SRC kinases and could be used for the development of new treatment strategies for ALL.

## Introduction

Acute lymphoblastic leukemia (ALL) is a type of cancer that leads to the proliferation of immature hematopoietic cells due to genetic alterations in lymphocyte precursors^1–6^. ALL is a heterogeneous disease that affects T- or B-lymphocyte precursors in 25 and 75% of cases, respectively. ALL accounts for approximately 12% of all cases of leukemia, which represents about one or two new events per 100,000 inhabitants per year. ALL mostly affects children aged two to five years, with 75% of patients under the age of eighteen. It accounts for approximately 30% of childhood cancers and for 80% of all leukemia cases in children. ALL also affects adults but to a lesser extent^7,8^. Thanks to improvements in the available treatments for ALL^1–6^, complete remission rates have become high, exceeding 80% in children and 50% in adults. However, long‐term survival at ten years (event‐free survival) is in the range of 60–80% for children and 25–35% for adults. This implies that there is still a significant need for new therapies to maintain remission and prolong survival. It is therefore necessary to improve our knowledge of this condition in order to discover new therapeutic strategies that would reduce the intensity of cytotoxic chemotherapy and improve the prognosis of patients after a relapse.

The chaperone Heat shock protein 90 (HSP90) plays a role in protecting the proper three-dimensional folding of proteins. HSP90 was found overexpressed in leukemia cells^9^, and its high expression was necessary for the survival and propagation of cancer cells. Treatments using HSP90 inhibitors that have been developed for solid tumors^10–13^ have therefore also been used for hematological disorders^14^. HSP90 inhibition has, for instance, been shown to be efficient for the treatment of lymphomas^15–17^. Several HSP90 inhibitors can overcome the resistance to Fms-like tyrosine kinase 3 (FLT3) inhibitors that has been observed in acute myeloid leukemia (AML)^18^. For the treatment of AML, the HSP90 inhibitor NVP-AUY922 has shown synergistic anti-leukemic activity with Cytarabine in vivo^19^, and Ganetespib (STA-9090) has also been tested in combination with Cytarabine as a potential active agent^20^. Alvespimycin (17-DMAG) administered intravenously twice weekly to AML patients was also found to be effective^21^. Co-treatments with 17-Allylamino-17-demethoxygeldanamycin (17-AAG) and FLT3 kinase or Histone deacetylase inhibitors were highly effective against human AML cells with mutant FLT3^22,23^. Elevated HSP90 inhibition disrupted JAK-STAT signaling and led to a reduction in splenomegaly in patients with myeloproliferative neoplasms^24^. HSP90 inhibitor was synergistic with JAK2 inhibitor and overcame resistance in human myeloproliferative neoplasm cells^25^. Other studies have also confirmed that there was an elevated expression of HSP90 in chronic myeloblastic leukemia (CML), suggesting that HSP90 could serve as a prognostic marker^26^. This also explains why several chemical inhibitors of HSP90 have been tested to treat CML^27^. In addition, targeting HSP90 dimerization was found effective in imatinib-resistant CML^28^.

Regarding ALL, the HSP90 inhibitor PU-H71 has been shown to be effective in treating T-ALL patients samples that express a high level of NOTCH1 (Notch receptor 1)^29^, NVP-AUY922 led to a degradation of Tyrosine kinase 2 (TYK2) signaling and T-ALL apoptosis^30^. In a subset of B-ALL, genetic resistance to Janus kinase 2 (JAK2) inhibition was overcome by HSP90 inhibition^31^. HSP90 expression in patients with T-ALL and B-ALL was significantly higher than those in a control group, and strong HSP90 expression was associated with a low survival rate^32^. Furthermore, plasmatic HSP90 has been validated as a soluble biomarker of T-ALL and B-ALL, which can be used for earlier detection of leukemia engraftment and progression in mice^33^.

SRC refers to a family of proto-oncogenes encoding the Lymphocyte-specific SRC family kinases (SFK). In this family, LCK (for Lymphocyte-specific protein tyrosine kinase) was highly expressed by T-ALL and was found essential for T-cell receptor (TCR) signaling^34,35^. Glucocorticoid resistance was reversed by LCK inhibition in pediatric T-ALL^36^. The inhibition of LCK, by preventing its phosphorylation, was an important strategy for the treatment of malignant hematopoiesis such as T-ALL, particularly with the use of Bosutinib, Dasatinib, or Saracatinib, which affected the proliferation of leukemia cells^36–38^. Its homolog protein LYN (Lck/Yes-related novel protein tyrosine kinase) was more specifically expressed by B-ALL and was important for B-cell receptor (BCR) signaling^39,40^. The inhibition of LYN was an important strategy for the treatment of B-ALL, more particularly with Dasatinib^41–43^. However, Ibrutinib inhibited BCR positive B-ALL progression by targeting important kinases in the BCR pathway^44^. In leukemia, HSP90 has been shown to bind to LYN in B-chronic lymphoblastic leukemia (B-CLL) and the use of 17-AAG destabilized the binding of HSP90-LYN in vitro, initiating cell apoptosis^45^.

Studies have revealed interactions of LCK^46^ and LYN^47^ with HSP90. The purpose of this project was first to test several HSP90 inhibitors in order to study their ability to deactivate the SFK clients of HSP90 in ALL. While HSP90 inhibitors were often investigated as anti-cancer drugs, we discovered that NVP-BEP800, which acts as an inhibitor of the ATP pocket of HSP90β^48^, can inhibit LCK in T-ALL and LYN in B-ALL. Also, we found that this drug reduced the viability of primary T-ALL and B-ALL cells in vitro. In addition, leukemia cell development and proliferation were inhibited in NVP-BEP800 treated xenografted mouse models.

## Materials and methods

### Patient samples

T-ALL and B-ALL samples, isolated from bone marrow (BM) or peripheral blood (PB) were collected from two independent cohorts in Dijon and Paris. For the first cohort, patients were included at diagnosis or relapse after giving their informed consent (Hôpital Universitaire François Mitterrand, CRB Ferdinand Cabanne, Dijon, France), under the reference number BB-0033-00044, in accordance with the declaration of Helsinki and under clinical trial reference nct04437420. Patients from the second cohort were children or young adults. Samples were included at diagnosis or relapse from the pediatric hematological unit (Dr. Paola Ballerini) at the Assistance Publique Hôpitaux de Paris (APHP, Paris, France), under the reference number CAALL-F01, in accordance with the declaration of Helsinki. Translocations, intrachromosomal deletions, and mutations in T-ALL and B-ALL were identified following specific procedures. The parents or representatives of patients younger than 18 years old gave informed consent.

### Establishment of xenograft models

The ethics committee for animal welfare of the University of Burgundy and the French ministry of higher education and research approved all animal experiments (under reference APAFIS#16187-2018071914379464v3). We confirm that all experiments were performed according to the relevant guidelines and regulations of this committee. NOD/SCID/γc^−/−^ (NSG) mice (Charles River) were bred and housed in pathogen-free conditions. Regarding cytogenetic characterization, T-ALL cells transplanted into PDX mice contain a STIL (*SCL/TAL1* interrupting locus), as well as deletions in *LEF1* and *CDKN2A* genes. Transplanted B-ALL cells, displayed a translocation t(2;8) (p11;q24) *MYC/IGK*. To induce leukemia in mice, we injected 10^5^ T-ALL or B-ALL cells in a volume of 300 µl of PBS1×, into the tail vein (intravenous; i.v.) of non-irradiated 7-16-week-old male and female NSG. Mice were treated with NVP-BEP800 (SelleckChem) at 10 mg/kg, with three i.v. injections on the days indicated on the figures. NVP-BEP800 was reconstituted in 100% ethanol at 10 mg/ml and diluted in 300 µl of PBS1× just before it was injected into the mice. Ethanol was the diluent, which served as control “vehicle” in vivo. Mice were randomly allocated to experimental groups and no blinding method was followed for injections. For experiments, we used males and females. There were no animal exclusion criteria. Mice were euthanized when moribund or at the indicated time points. After tail vein PB sampling, hematopoietic cells were counted using a hemocytometer (Vet ABC+, SCIL).

### Bioluminescence imaging

We created PDX models that developed stable bioluminescence by infecting T-ALL and B-ALL cells with a lentivirus expressing both GFP and luciferase. Then, we transplanted these cells into mice that were used later to perform bioluminescence imaging. The lentivirus was produced in HEK293 cells after transduction with Lipofectamin 2000 (Thermo Fisher Scientific) of the pCCLc-MNDU3-Luciferase-PGK-EGFP-WPRE vector (Addgene, #89608), as well as PAX2 (Addgene, #12260) and pCMV-VSV-G (Addgene, #8454) plasmids. After two days, viral supernatants were recovered, and six-well plates were incubated 4 h with retronectin (Takara, Ozyme). Viral supernatants were then spinoculated for 30 min at 4,000 g. Cells were cultured on these plates for three days in StemMACS media (Miltenyi Biotech). Lentiviral transduced cells (GFP^+^) were sorted on a FACSAriaIII cell sorter (BD Biosciences) and transplanted in NSG mice to generate bioluminescent PDX models. Animals were injected with potassium salt of D-luciferin (150 mg/kg body weight). Following isoflurane-induced anesthesia, animals were imaged 20 min after D-luciferin injection using an IVIS Lumina III system coupled to Living Image acquisition and analysis software version 4.0 (Perkin Elmer).

### Statistics

All data were expressed as means ± standard deviation (SD). Differences between two groups were assessed with the two-tailed unpaired Student’s *t* test, two-tailed paired Student’s *t* test or the Wilcoxon–Mann–Whitney test. The one-way Anova with Tukey’s multiple comparison test was used to assess differences between more than two groups. Survival curves were assessed using the Mantel–Haenszel (Log-Rank) test. No statistical methods were used to predetermine the sample size. The variance was similar between the groups that were statistically compared. Statistics were performed using Prism 6 (GraphPad), where significance is indicated on the figures.

Cell culture and treatment with NVP-BEP800, cell viability assay (XTT), western blot, immunoprecipitation, flow cytometry, fluorescent-activated cell sorting (FACS), fluorescence microscopy, immunohistochemistry, quantitative reverse transcription PCR, shRNA lentiviral cloning and viral infection, as well as high-performance liquid chromatography (HPLC) were performed as described in the supplementary materials and methods.

## Results

### NVP-BEP800 affects viability of lymphoid lines expressing SRC

**Fig. 1:**
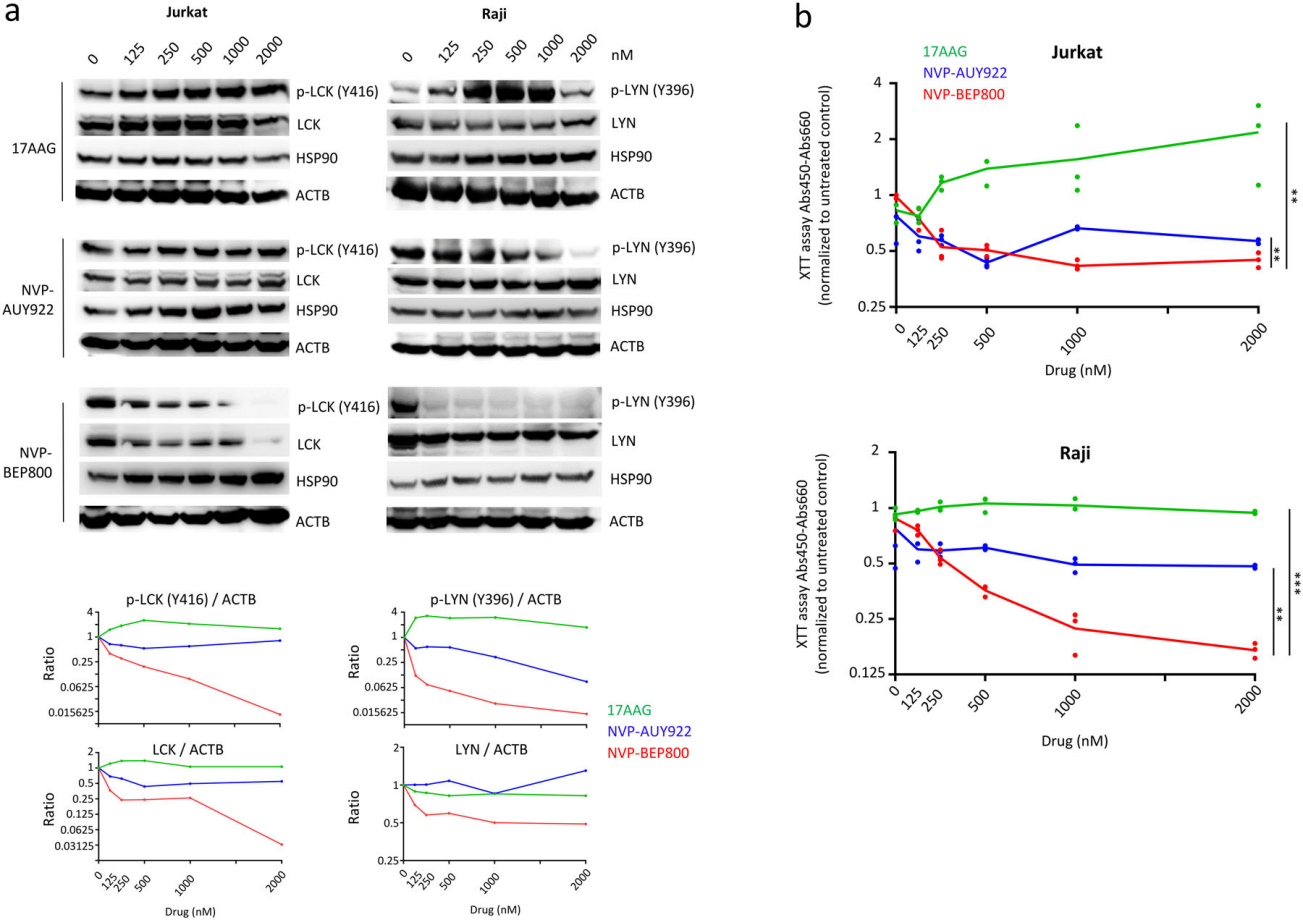
Sensitivity of T- and B-lymphoid cell lines to NVP-AUY922, 17-AAG and NVP-BEP800. **a** Western blot shows that in Jurkat and Raji cells, levels of phosphorylated SRC and total SRC proteins are more affected after treatment with NVP-BEP800, compared to NVP-AUY922 or 17-AAG. Western blots after 18 h of treatment are shown on the top panel and quantifications on the bottom panel. **b** XTT viability assay after 48 h of treatment shows that Jurkat and Raji cells are more sensitive to NVP-BEP800, compared to the other two compounds. Data are shown as mean; *n* = 3 biological replicates. *P* value measured by one-way Anova test with Tukey’s multiple comparison test; **P < 0.01; ***P < 0.001.

HSP90 (Heat shock protein 90) is a chaperone protein that modulates intracellular signaling and protein folding. It also stabilizes several other proteins implicated in tumor growth. Lymphocyte-specific SRC family kinases (SFK) are important regulators of pathways involved in the proliferation and growth of lymphoid leukemia cells. Our aim was therefore to test whether HSP90 inhibitors had an effect on the stability of SRC proteins. We focused on inhibitors that target the N-terminal ATP-binding pocket of HSP90 rather than the C-terminal portion, since they were more potent inhibitors^11^. We tested two compounds that target both HSP90α and HSP90β, Luminespib (NVP-AUY922)^49^ and 17-AAG^50^. We also tested NVP-BEP800, an inhibitor that was discovered to target only HSP90β^48^. Among the SFK, T-cells expressed more LCK^51^, while B-cells expressed more LYN^40^. When we examined the effect of the three compounds on the stability of phosphorylated SRC (active form) and the total amount of SRC proteins, NVP-BEP800 was the most efficient (Fig. 1a). Furthermore, loss of LCK and LYN was observed between 12 and 24 h after the treatment of Jurkat or Raji cells on a time-course experiment (Supplementary Fig. S1). Using the XTT assay to study the viability, we found that ALL cells were more sensitive to NVP-BEP800, than the other two compounds (Fig. 1b). We next used two T-ALL cell lines, the Jurkat line expressing LCK and the Rpmi-8402 line that showed no expression of LCK^51^. Through western blot, NVP-BEP800 was found to affect the stability of phosphorylated LCK and the total amount of LCK in the Jurkat line, while both cell lines were expressing HSP90 (Supplementary Fig. S2a). The XTT assay showed that cells that expressed more LCK (Jurkat) were more sensitive (*P* < 0.001) to NVP-BEP800, compared to non-expressing cells (Rpmi-8402) (Supplementary Fig. S2b). Using four B-lymphoid cell lines (Raji, Daudi, Reh, and BALL-1), western blot demonstrated different levels of phosphorylated LYN and total amounts of LYN, and the protein levels could be affected by NVP-BEP800 treatment (Supplementary Fig. S2c). With the XTT assay, we also observed that, after NVP-BEP800 treatment, the sensitivity of these cell lines was correlated with the quantity of p-LYN measured by western blot (*R* = 0.979) (Supplementary Fig. S2d). All cell lines expressed the HSP90 chaperone, indicating that sensitivity to the compound was correlated only with p-LYN expression. In addition, NVP-BEP800 which is an ATP-competitive inhibitor specific for HSP90β^48^ did not affect the expression levels of neither HSP90α nor β isoforms. Also, NVP-BEP800 did not affect the protein level of the HSP70 chaperone and there was no effect on the BCL2 protein, which is involved in apoptosis (Supplementary Fig. S3).

In conclusion, using T- and B-lymphoid cell lines, we observed that NVP-BEP800, a specific inhibitor of HSP90β, affected the stability of SRC kinases, making them potential clients of the HSP90 protein in lymphoid leukemic cells.

### Knockdown of SRC affects response of lymphoid lines to NVP-BEP800

**Fig. 2:**
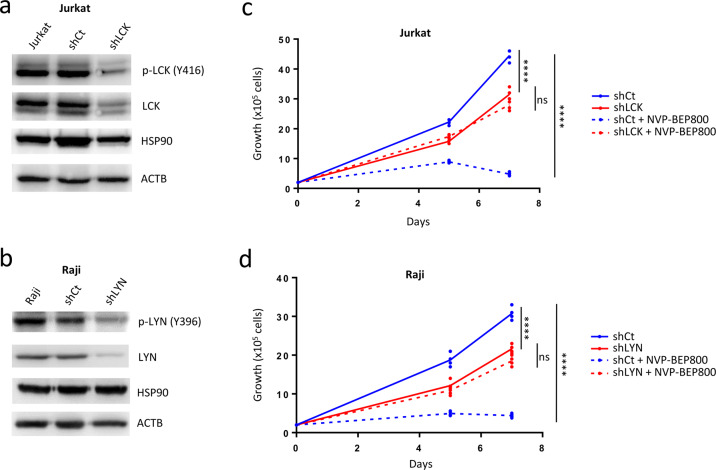
Downregulation of LCK and LYN kinases inhibits NVP-BEP800 efficiency on T and B lymphoblast cell lines. Western blot shows low levels of expression of p-LCK and total LCK after the viral transduction of Jurkat cells with shLCK (**a**), or p-LYN and total LYN after the viral transduction of Raji cells with shLYN (**b**). Cell growth assay shows no sensitivity of shLCK Jurkat cells (**c**) and shLYN Raji cells (**d**) to NVP-BEP800. 2 × 10^5^ viable cells (trypan blue negative) are seeded. Cells are treated with NVP-BEP800 (1 µM) at days 0 and 5. Data are shown as mean; *n* = 3 biological replicates for T-ALL and *n* = 4 biological replicates for B-ALL. *P* value measured by one-way Anova test with Tukey’s multiple comparison test; ****P < 0.0001; ns, non-significant.

We knocked down LCK or LYN genes’ expression, respectively in Jurkat and Raji cells using specific shRNA (shLCK or shLYN), throughout lentiviral infection (Supplementary Figs. S4a and S5a) and observed by western blot specific downregulation of LCK (Fig. 2a) or LYN (Fig. 2b). Specificity of the shRNA for LCK or LYN over other tyrosine kinases was furthermore confirmed (Supplementary Figs. S4b and S5b). Both shLCK and shLYN cells showed a reduction in the percentage of cells in the active phase of division (Ki67^+^ 7-AAD^+^) and more cells underwent apoptosis (Annexin-V^+^) (Supplementary Figs. S4c and S5c). When we treated shLCK cells or shLYN cells with NVP-BEP800, they showed a significant loss of sensitivity to the compound, 48 h after treatment, compared to shCt control cells (*P* < 0.0001), as measured by XTT viability assay (Supplementary Figs. S4d and S5d). We then analyzed cell growth in vitro during 7 days, shLCK Jurkat cells (Fig. 2c) and shLYN Raji cells (Fig. 2d) showed reduced growth capacity compared to their shCt control cells. Since NVP-BEP800 can target several other clients’ proteins^11,52^ which went beyond the inhibitory effect of BEP800 on the SRC family of SFK, thus, shLCK Jurkat and shLYN Raji cells treated with NVP-BEP800 showed, albeit slightly, a decrease in growth rate compared to the untreated cells (Fig. 2c, d). However, after treatment with NVP-BEP800, while shCt controls cells were very sensitive to treatment (*P* < 0.0001), shLCK Jurkat cells (Fig. 2c) and shLYN Raji cells (Fig. 2d) were insensitive (*P* > 0.05).

In conclusion, the use of shRNA to knock down LCK or LYN provided evidence that the cytotoxic effects of NVP-BEP800 were mediated by the degradation of SRC proteins.

### Sensitivity of primary ALL samples to NVP-BEP800 correlates with expression of SRC

**Fig. 3:**
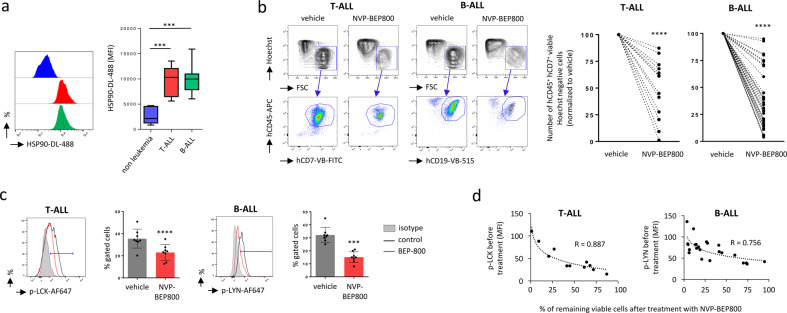
Primary T-ALL and B-ALL cells are sensitive to NVP-BEP800. **a** Flow cytometry on primary T-ALL (CD45^+^ hCD7^+^, *n* = 10), B-ALL (hCD45^+^ CD19^+^, *n* = 29) and non-leukemic (hCD45^+^, *n* = 6) shows elevated expression of HSP90 for ALL cells. Examples of median fluorescence intensity (MFI) observed by flow cytometry and statistics. Data are presented as median (central line), first and third quartiles (bottom and top of boxes, respectively), and whiskers (extreme values). *P*-value measured by Wilcoxon-Mann-Whitney test; ***P < 0.001. **b** Flow cytometry of representative primary ALL cells treated with NVP-BEP800 (1 µM, for 48 h) shows a decrease in the leukemic cell count of T-ALL cells (hCD45^+^ hCD7^+^ cells) and of B-ALL cells (hCD45^+^ hCD19^+^). Examples of cytometry dataset are shown on the left panel, data are gated on viable Hoechst negative cells. Statistics is shown on the right panel. For quantification, primary cells are fully recorded by flow cytometry. The number of viable primary cells in the group of cells treated with NVP-BEP800 is normalized to control vehicle, for T-ALL (*n* = 13) and B-ALL (*n* = 39). Data are shown as mean ± SD. *P*-value measured by two-tailed paired Student’s *t* test; ****P < 0.0001. **c** Flow cytometry of representative primary ALL cells treated with NVP-BEP800 (1 µM, for 18 h) shows a reduction of p-LCK in T-ALL (*n* = 8) and p-LYN in B-ALL (*n* = 8) cells. Data are gated on viable Hoechst negative cells, hCD7^+^ (T-ALL) or hCD19^+^ (B-ALL). Data are shown as mean ± SD. *P*-value measured by two-tailed paired Student’s *t* test; ***P < 0.001; ****P < 0.0001. **d** The percentage (%) of viable primary cells after treatment with NVP-BEP800 is correlated with the expression of p-LCK or p-LYN, measured by flow cytometry before the treatment, for T-ALL (*n* = 11) and B-ALL (*n* = 23). The logarithmic correlation coefficient R is reported. MFI; median fluorescence intensity.

The levels of HSP90 expression in flow cytometry in primary cells isolated from the bone marrow (BM) or peripheral blood (PB) of patients diagnosed with T- or B-ALL was higher than in hematopoietic cells (CD45^+^) isolated from patients diagnosed with hematological disorders other than ALL (e.g. anemia or thrombocytopenia) (Fig. 3a). We tested the efficiency of the NVP-BEP800 on primary ALL cells in vitro, and observed that both T-ALL cells (hCD45^+^ hCD7^+^ cells) and B-ALL cells (hCD45^+^ hCD19^+^) were sensitive to the compound, and 2 days after the treatment, a reduction in viability was observed for the 13 T-ALL samples (*P* < 0.0001) and 39 B-ALL samples (*P* < 0.0001) (Fig. 3b). Primary T-ALL cells showed specific expression of LCK, while primary B-ALL cells expressed more LYN (Supplementary Fig. S6). Flow cytometry of primary ALL cells treated with NVP-BEP800 showed a reduction in tyrosine phosphorylation of LCK (p-LCK) in T-ALL cells (*P* < 0.0001) and tyrosine phosphorylation of LYN (p-LYN) in B-ALL cells (*P* < 0.001) (Fig. 3c). We observed that T-ALL and B-ALL cells expressing high levels of p-LCK or p-LYN were more sensitive to NVP-BEP800, and we also noted a correlation (*R* = 0.887 for T-ALL and *R* = 0.756 for B-ALL cells) between the rates of p-LCK or p-LYN measured by flow cytometry before treatment and the percentage of remaining viable cells after treatment (Fig. 3d). No correlation was observed with genetic alterations, and there was no difference in sensitivity detected between children or adults with ALL. However, we observed that sensitivity could correlate with the stage of B-ALL maturation (Supplementary Figs. S7 and S8). We found no correlation between sensitivity to the compound and expression of HSP90 measured by flow cytometry (Supplementary Fig. S9a). Moreover, we found no correlation between the expression levels of HSP90 and SFK proteins (Supplementary Fig. S9b).

In conclusion, primary T-ALL and B-ALL samples showed sensitivity to NVP-BEP800, and this sensitivity was related to their expression of SFK.

### NVP-BEP800 affects the SRC signaling pathway in ALL cells recovered from PDX mice

**Fig. 4:**
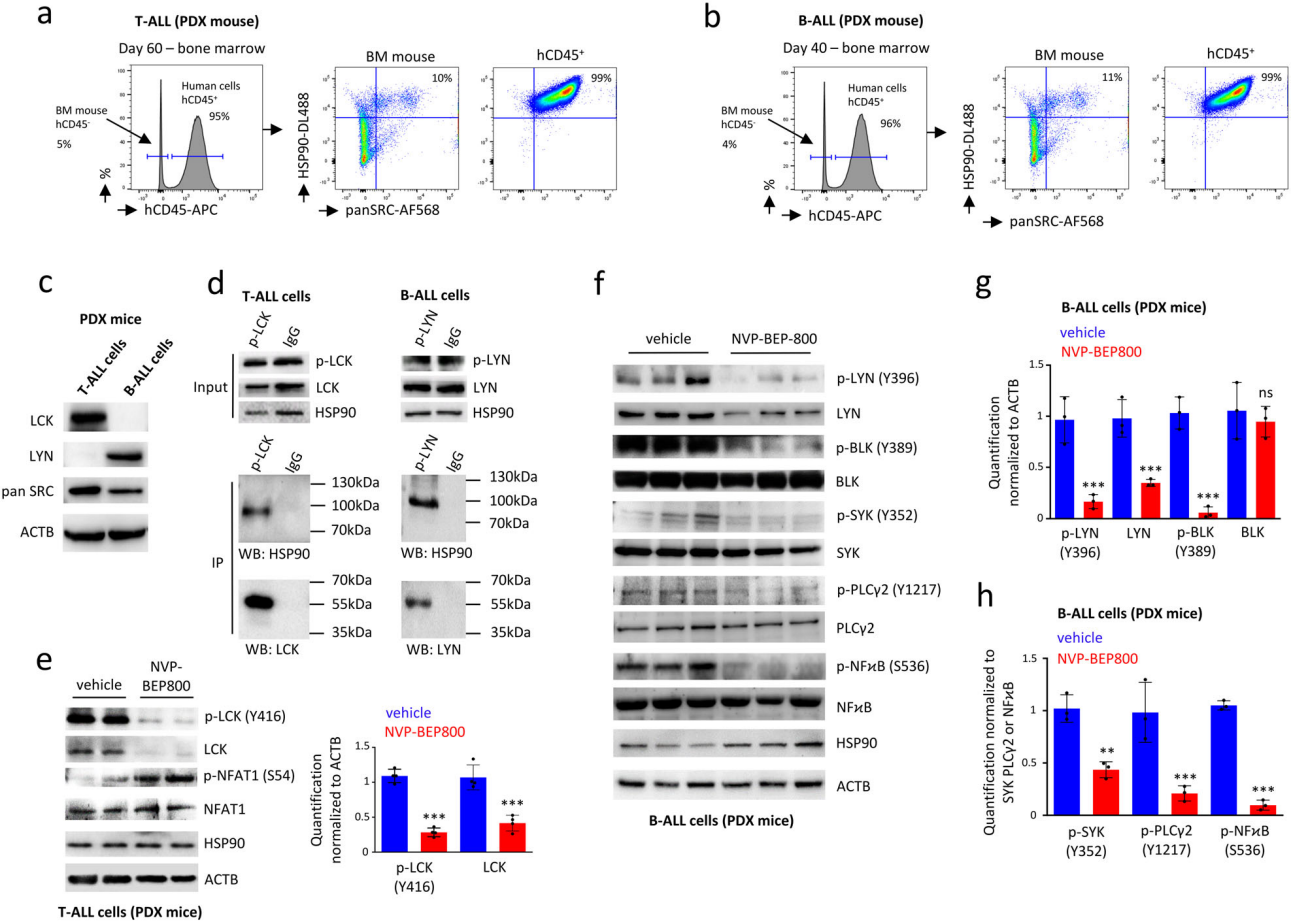
SRC kinases are client proteins of HSP90. Flow cytometry on BM cells from PDX models of T-ALL (**a**) and B-ALL (**b**), demonstrates that ALL (hCD45^+^) cells express a high level of HSP90 and panSRC. The anti-panSRC antibody detects murine and human panSRC family kinases, and the anti-HSP90 antibody detects murine and human HSP90. **c** Western blot on ALL cells recovered from PDX mice, shows a high level of expression of LCK in T-ALL, while B-ALL expresses more LYN. **d** Immunoprecipitation shows that LCK binds to HSP90 in PDX T-ALL cells and LYN binds to HSP90 in PDX B-ALL cells. **e** Treatment of PDX T-ALL cells ex vivo for 18 h with NVP-BEP800 at 1 µM affects the stability of the phosphorylated LCK as well as total LCK. The treatment inhibits the transcription factor NFAT1 that becomes phosphorylated. Data are shown as mean ± SD; *n* = 4 biological replicates. *P*-value measured by two-tailed unpaired Student’s *t* test; ***P < 0.001. **f** Western blot shows that treating PDX B-ALL cells with NVP-BEP800 for 18 h, affects the stability of phosphorylated LYN and total LYN. The downstream BCR pathway involving phosphorylation of BLK, SYK, PLCγ2, and NFϰB is also affected. **g** Quantification of the western blot shows that among BLK and LYN kinases, only the total amount of LYN is affected following treatment. **h** Quantification of the western blot shows that phosphorylation of SYK, PLCγ2, and NFϰB is affected following treatment. Data are shown as mean ± SD; *n* = 3 biological replicates. *P*-value measured by two-tailed unpaired Student’s *t* test; **P < 0.01; ***P < 0.001; ns, non-significant.

Through the transplantation of primary ALL cells into immunodeficient NSG mice, we generated PDX models to study T-ALL and B-ALL in vivo. By flow cytometry, we detected major expressions of HSP90 and SRC in ALL cells recovered from the BM of T-ALL (Fig. 4a) and B-ALL PDX mice (Fig. 4b). HSP90 is known to regulate the stability of proteins involved in intracellular signaling. Interestingly, fluorescence microscopy revealed that SRC and HSP90 proteins were colocalized in the cytoplasm of T-ALL (*R* = 0.91 ± 0.07) and B-ALL cells (*R* = 0.89 ± 0.06) that were recovered ex vivo from the BM of PDX mice (Supplementary Fig. S10). Among the SFK, T-ALL cells expressed more LCK, while B-ALL expressed more LYN, which was confirmed by western blot (Fig. 4c). Previous studies revealed that HSP90 can interact physically with LCK and LYN^46,47^. When SRC kinases were pulled down with specific antibodies, HSP90 was found co-immunoprecipitated in the T-ALL and B-ALL cell lysates, confirming an interaction between HSP90, with LCK in T-ALL and LYN in B-ALL (Fig. 4d).

When cells isolated ex vivo from T-ALL PDX mice were treated with NVP-BEP800, we observed a loss of LCK phosphorylation in its active site (on tyrosine 416) and a loss in the total amount of LCK protein (*P* < 0.001) (Fig. 4e). This kinase, through the calcium influx pathway, was found specific to the regulation of Nuclear factor of activated T cell 1 (NFAT1), which was involved in T-ALL cell survival and proliferation^38^. When inactivated, NFAT1 was phosphorylated (on Serine 54) (Fig. 4e) and left the nucleus to reach the cytoplasm (Supplementary Fig. S11a). Regarding B-ALL cells isolated ex vivo from PDX mice and treated with NVP-BEP800, we observed a loss of LYN phosphorylation in its active site (on tyrosine 396), and a loss in the total amount of LYN protein (*P* < 0.001) (Fig. 4f, g). In B-cells, LYN contributed to positive regulation of signaling through tyrosine phosphorylation of the BCR. This role can be assumed by B-lymphocyte kinase (BLK), which can promote B-cells activation through the recruitment of Spleen tyrosine kinase (SYK)^40^. The protein tyrosine kinases, such as LYN, SYK and BLK, and effector enzymes, such as Phospholipase Cγ2 (PLCγ2), played a crucial role in the BCR-induced activation of Nuclear factor κB (NFϰB), which was important for the outcome of B-cells^53^. After treatment with the NVP-BEP800 inhibitor, we observed substantial deregulation of the entire signaling pathway, as suggested by the reduced phosphorylation of BLK, SYK, PLCγ2, and NFϰB observed with western blot (Fig. 4f, g, h). Upon inactivation, phosphorylation (on Serine 536) was lost and NFϰB left the nucleus to reach the cytoplasm (Supplementary Fig. S11b).

We can therefore conclude that the HSP90 chaperone bound SRC kinases in ALL cells and that inhibition of HSP90 through the use of the chemical compound NVP-BEP800 has affected the downstream SRC signaling pathways involved in the proliferation and growth of T-ALL and B-ALL cells.

### NVP-BEP800 affects cell cycle and induces apoptosis of ALL cells

**Fig. 5:**
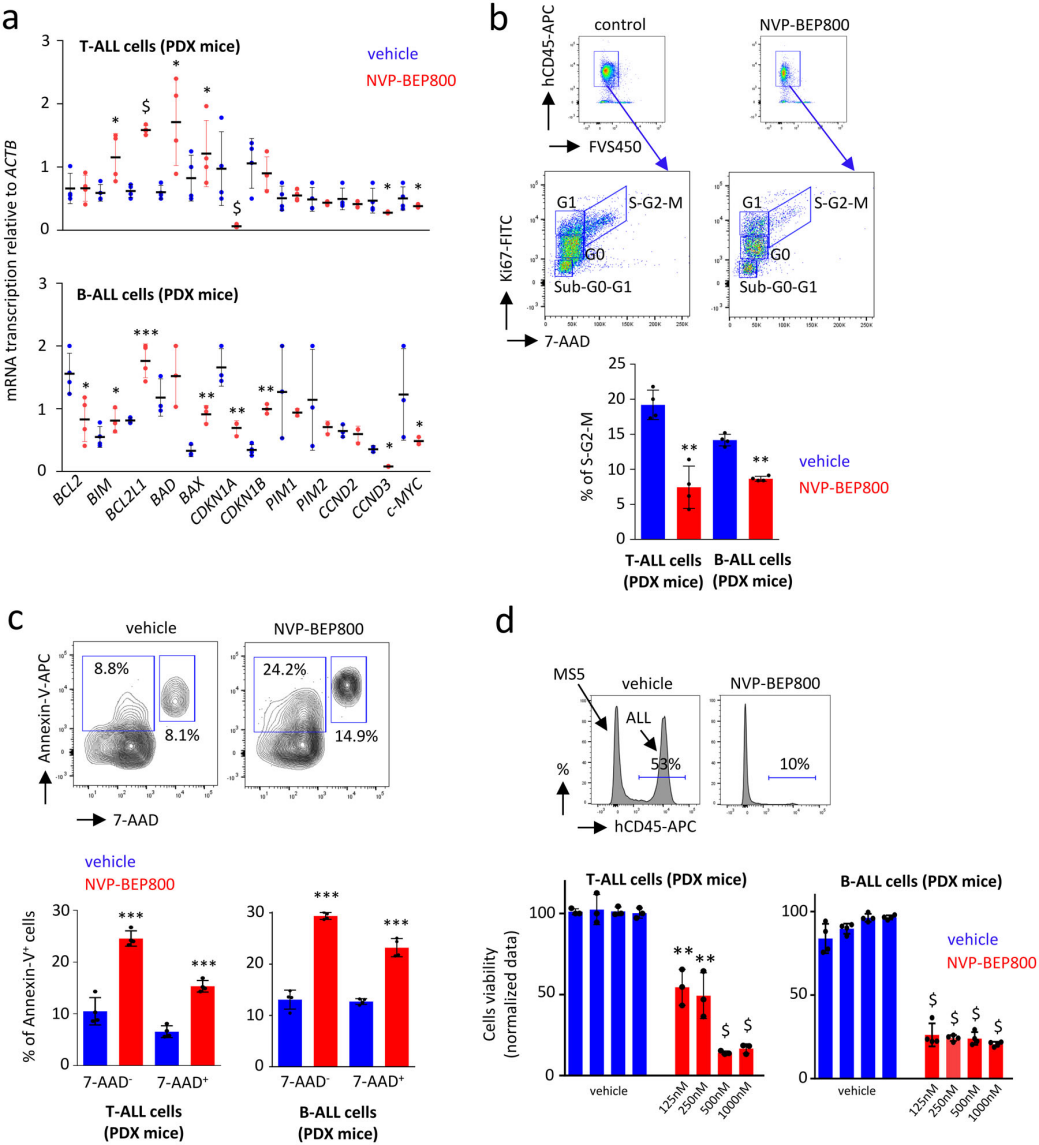
NVP-BEP800 affects the viability of T-ALL and B-ALL cells. **a** RTqPCR, performed on T-ALL and B-ALL cells, shows modification in the transcription of genes involved in cell cycle and apoptosis, after treatment with NVP-BEP800 (1 µM) within 18 h. Data are shown as mean ± SD (biological replicates). *P*-value measured by two-tailed unpaired Student’s *t* test; *P < 0.05; **P < 0.01; ***P < 0.001; $, *P* < 0.0001; data without statistic are non-significant. **b** A cell cycle study performed by Ki67 staining. **c** An apoptosis study performed by Annexin-V staining. Data shows early apoptotic cells (7-AAD^-^ Annexin-V^+^) and late apoptotic cells (7-AAD^+^ Annexin-V^+^). Flow cytometry performed on T-ALL or B-ALL cells isolated from BM and treated ex vivo with NVP-BEP800 (1 µM) within 18 h. Data are shown as mean ± SD; *n* = 4 biological replicates. *P*-value measured by two-tailed unpaired Student’s *t* test; **P < 0.01; ***P < 0.001. **d** T-ALL (*n* = 3 biological replicates) and B-ALL (*n* = 4 biological replicates) cells are cultured on MS5 support cells for 48 h, under treatment with NVP-BEP800 at increased concentrations (from 125 to 1,000 nM). Ethanol is the vehicle used as control. Flow cytometry is used to quantify ALL cells (hCD45^+^) after two days of treatment. Data are shown as mean ± SD. *P*-value measured by two-tailed unpaired Student’s *t* test; **P < 0.01; $, *P* < 0.0001.

SRC kinases were involved in signaling pathways necessary for survival, growth, and maintenance of T-ALL^36–38^ and B-ALL cells^41–43^. To confirm that NVP-BEP800 has an effect on the viability of T-ALL or B-ALL cells recovered from PDX mice, we analyzed the transcription of several genes involved in the cell cycle and apoptosis after treatment (Fig. 5a). NVP-BEP800 increased the transcription of the pro-apoptotic genes *BCL2L1*, *BAD*, *BAX*, and *BIM*, and decreased the transcription of *CDKN1A*, a negative regulator of cell levels of p53. Furthermore, treatment with NVP-BEP800 induced the downregulation of *CCND3* and *c-MYC* genes transcription, which are both involved in the cell cycle. Ki67 staining and flow cytometry revealed a marked reduction of T-ALL or B-ALL cells in division (mitosis), following treatment with NVP-BEP800, as demonstrated by the low percentage of cells in the S-G2-M phase (Fig. 5b). Annexin-V staining and flow cytometry showed an increase in the percentage of T-ALL and B-ALL cells undergoing apoptosis after NVP-BEP800 treatment (Fig. 5c), which was furthermore confirmed by increased levels of cleaved Caspase-3 after treatment (Supplementary Fig. S12). When T-ALL and B-ALL cells were cultured on MS5 murine stromal cells for support, we found that the viability of leukemic cells was significantly affected by this treatment (Fig. 5d).

In conclusion, NVP-BEP800 has affected the viability of T-ALL and B-ALL cells ex vivo by dysregulating the SRC kinases involved in cell proliferation and survival.

### Activation of LCK or LYN antagonizes the inhibitory effect mediated by NVP-BEP800 on T-ALL and B-ALL cells

**Fig. 6:**
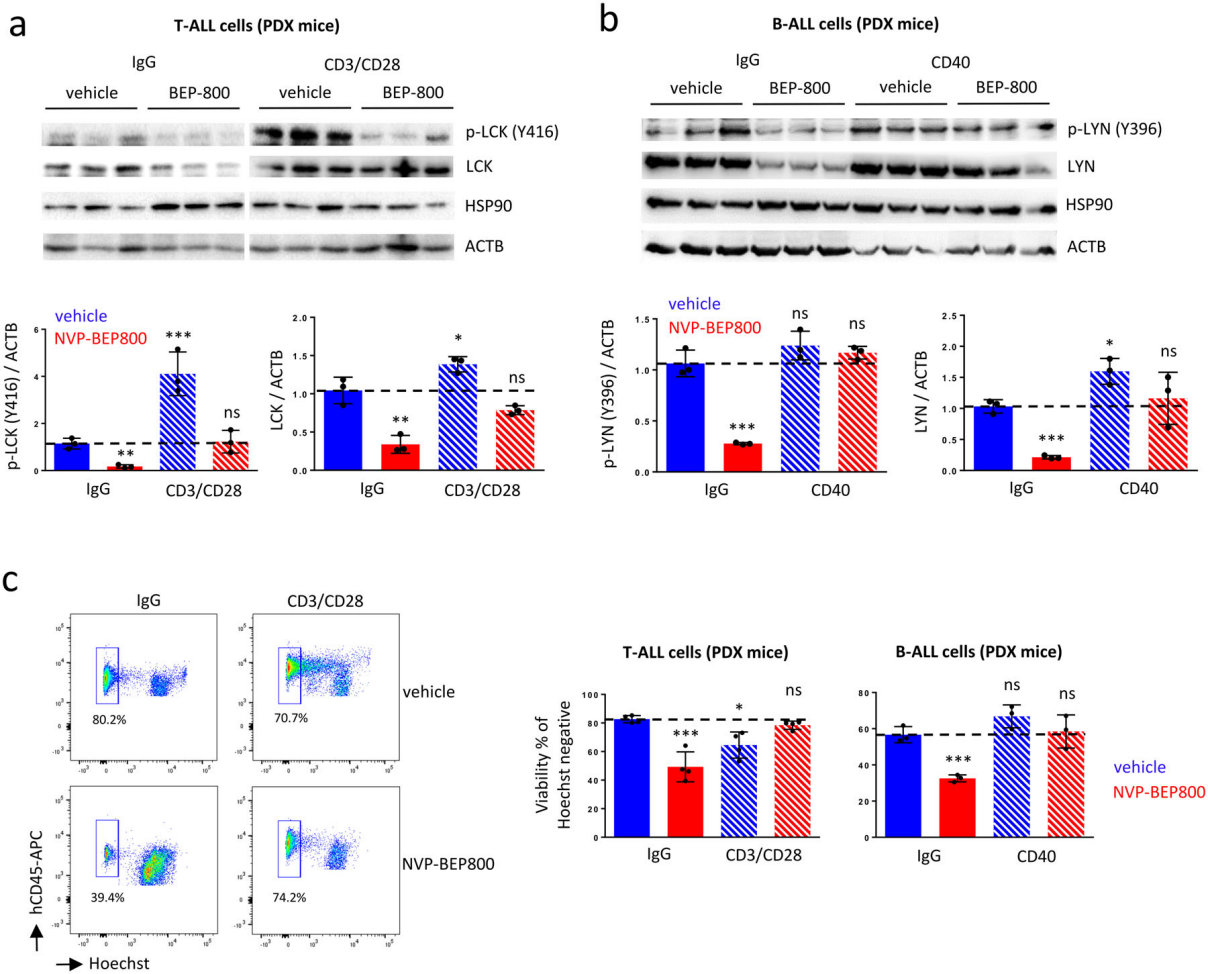
Activation of LCK or LYN antagonizes the inhibitory effect mediated by NVP-BEP800 on T-ALL and B-ALL cells isolated from PDX mice. **a** Western blot shows that cross-linking with anti-CD3 (1 μg/ml) and anti-CD28 (1 μg/ml) antibodies (CD3/CD28) to activate the LCK kinase inhibits the effect of NVP-BEP800 and its capacity to reduce levels of p-LCK and LCK. Quantification of p-LCK (Y416) and LCK is normalized to ACTB (bottom panel). Data are shown as mean ± SD; *n* = 3 biological replicates. **b** Activation of the LYN kinase antagonizes the effect of NVP-BEP800. Western blot on PDX B-ALL cells pretreated or not with anti-CD40 (1 μg/ml) antibody (CD40). Quantification of p-LYN (Y396) and LYN is normalized to ACTB (bottom panel). Data are shown as mean ± SD; *n* = 3 biological replicates. **c** Cytometry shows that activation of LCK or LYN antagonizes the inhibitory effect mediated by NVP-BEP800, on T-ALL and B-ALL PDX cells. After treatment with anti-CD3 (1 μg/ml) and anti-CD28 (1 μg/ml) antibodies (CD3/CD28) to activate the LCK kinase or anti-CD40 (1 μg/ml) antibody (CD40) to activate the LYN kinase, ALL cells are treated with NVP-BEP800 (1 µM) for two days and the viability of ALL cells is recorded by flow cytometry after Hoechst staining. Data are shown as mean ± SD; *n* = 4 biological replicates for T-ALL and *n* = 3 biological replicates for B-ALL. In this figure, *P*-value measured by one-way Anova test with Tukey’s multiple comparison test; IgG vehicle is used as a reference; *P < 0.05; **P < 0.01; ***P < 0.001; ns, non-significant.

SRC kinases were important regulators of TCR and BCR receptors^54^. To confirm the implication of the LCK kinase as the main client of HSP90 in T-ALL, we over activated the TCR pathway via anti-CD3/CD28 monoclonal antibodies. T-ALL cells expressed CD3 and CD28 on the cell surface, as assessed by flow cytometry (Supplementary Fig. S13a). Cross-linking of CD3/CD28 antagonized the ability of NVP-BEP800 to induce complete loss of p-LCK and LCK (Fig. 6a). Signal transduction via CD40 involved activation of LYN kinase and PLCγ2 in B-cells^55^, and when these cells were activated via anti-CD40 antibody in vitro they underwent survival^56^. Using flow cytometry, we showed that B-ALL cells expressed CD40 on the cell surface (Supplementary Fig. S13b). On western blot, we found that the cross-linking of CD40 has inhibited the ability of NVP-BEP800 to induce a loss of p-LYN and LYN (Fig. 6b). Although CD3/CD28 stimulated T-lymphocyte proliferation in vitro^57^, it induced the apoptosis of T-ALL cells^58^. We observed a slight decrease in T-ALL viability with CD3/CD28; however, the negative effect of NVP-BEP800 on the viability of T-ALL cells was antagonized after cross-linking of CD3/CD28 (Fig. 6c). Additionally, the effect that NVP-BEP800 had on the viability of B-ALL cells was also antagonized after cross-linking of CD40 (Fig. 6c).

In conclusion, we found that an over-activation of the SRC kinase pathways limited the effect of NVP-BEP800 on SRC stability as well as ALL viability, confirming that this compound has affected the SRC signaling pathway involved in the growth of ALL cells.

### NVP-BEP800 increases survival of PDX mice developing T-ALL or B-ALL

By flow cytometry, we detected major expressions of HSP90 and SRC for T-ALL and B-ALL cells recovered from the BM of PDX mice, when they were compared to normal murine cells in a BM microenvironment in which leukemic cells were engrafted and expanded (Fig. 4a, b). Therefore, targeting the HSP90 is a good strategy to prevent T-ALL and B-ALL growth in vivo.

**Fig. 7:**
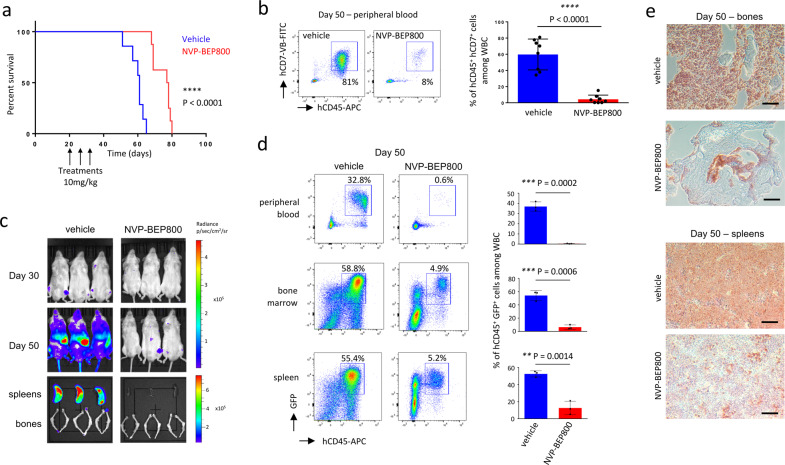
Treatment with NVP-BEP800 improves the survival of T-ALL PDX mice. **a** Survival curves show that mice treated with NVP-BEP800 (10 mg/kg) survived longer than mice treated with vehicle, *n* = 8 mice per group. *P* value measured by Mantel Haenszel test. The timing of treatment is shown on the graphic. **b** Percentage of T-ALL cells (hCD45^+^ hCD7^+^) is measured by flow cytometry in PB at day 50 of the study, *n* = 8 mice per group. Data are shown as mean ± SD. *P*-value measured by two-tailed unpaired Student’s *t* test; ****P < 0.0001. **c** Mice are transplanted with bioluminescent T-ALL cells and treated with NVP-BEP800. Bioluminescence images (*n* = 3 mice) per group, are taken at days 30 and 50 post-transplantation of bioluminescent T-ALL cells. At day 50, mice are sacrificed and bioluminescence images of their spleens and bones are taken. **d** PB, spleen and BM are analyzed by flow cytometry to detect bioluminescent leukemic cells also expressing GFP and stained with hCD45 antibody. Data shows a reduction of T-ALL cells in all organs in treated mice. Data are shown as mean ± SD; *n* = 3 mice. *P*-value measured by two-tailed unpaired Student’s *t* test; **P < 0.01; ***P < 0.001. **e** Immunohistochemistry on bones and spleens sections shows a decrease in the amount of T-ALL cells (brown cells) in mice treated with NVP-BEP800. Data are representative of three mice, magnification ×10, black scale bars represent 200 µm.

When NVP-BEP800 was injected intravenously (i.v.) at 10 mg/kg, we detected a concentration of 5 µM in the plasma as well as in BM, one hour after the injection (Supplementary Fig. S14), and this concentration was approximately five times the half maximal inhibitory concentration (IC50) observed in vitro. Based on this, we investigated to what extent NVP-BEP800 efficiently interfered with leukemia progression in vivo. The first group of mice was injected i.v. with 10 mg/kg of NVP-BEP800 on day 20, 25, and 30 after the transplantation of 100,000 T-ALL cells, while the second group of mice was injected with the vehicle (ethanol). PDX mice treated with NVP-BEP800 survived longer than PDX mice treated with the vehicle (*P* < 0.0001, Fig. 7a). When leukemia progression was followed in PB at day 50 post-transplantation, we observed a reduced amount of leukemic cells (hCD45^+^ hCD7^+^) in PB of mice treated with NVP-BEP800 (*P* < 0.0001, Fig. 7b).

Additionally, we developed T-ALL cells that expressed luciferase and green fluorescence protein (GFP) using lentiviral infection of T-ALL cells, and we transplanted these bioluminescent T-ALL cells into mice. Again, the first group of mice was treated with 10 mg/kg of NVP-BEP800 and the second was injected with ethanol on day 20, 25, and 30 after the transplantation of 100,000 bioluminescent T-ALL cells. At day 30 and day 50, we injected luciferin into sleeping mice in order to monitor the location of leukemic cells in living animals, and we observed reduced bioluminescence in the group of mice treated with NVP-BEP800, the difference was evident at day 50 (Fig. 7c). Mice were sacrificed on day 50 post-transplantation. A decrease in bioluminescence was observed in spleens and bones isolated from mice in the treated group (Fig. 7c). When we performed flow cytometry to detect bioluminescent cells that express also GFP, we noted a relevant decrease in T-ALL cells in the PB, BM, and spleens of treated mice (Fig. 7d). Immunohistochemistry on BM and spleen sections stained with an hCD7 antibody revealed a more significant expansion of T-ALL cells in the control group, compared with mice in the treated group (Fig. 7e).

**Fig. 8:**
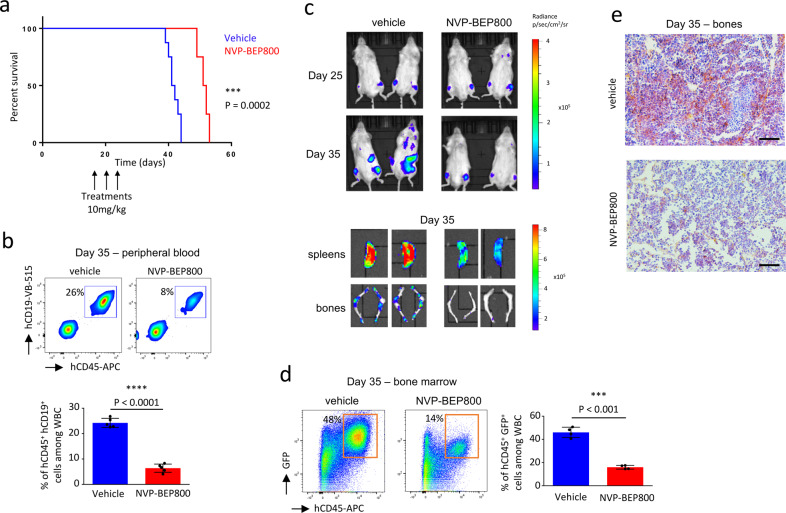
Treatment with NVP-BEP800 improves survival of B-ALL PDX mice. **a** Survival curves show that mice treated with NVP-BEP800 (10 mg/kg) survived longer than mice treated with vehicle, *n* = 8 mice per group. *P* value measured by Mantel–Haenszel test; ***P < 0.001. The timing of treatment is shown on the graphic. **b** Percentage of B-ALL cells (hCD45^+^ hCD19^+^) is measured by flow cytometry in PB at day 35 of the study, *n* = 5 mice per group. Data are shown as mean ± SD. *P*-value measured by two-tailed unpaired Student’s *t* test; ****P < 0.0001. **c** Mice are transplanted with bioluminescent B-ALL cells and treated with NVP-BEP800. Bioluminescence images of two mice per group, are taken at days 25 and 35 post-transplantation of bioluminescent B-ALL cells. At day 35, mice are sacrificed and bioluminescence images of their spleens and bones are taken. **d** Data show a reduction of B-ALL cells in BM of treated mice, as analyzed by flow cytometry to detect bioluminescent leukemic cells also expressing GFP and stained with hCD45 antibody. Data are shown as mean ± SD; *n* = 4 mice. *P*-value measured by two-tailed unpaired Student’s *t* test; ***P < 0.001. **e** Immunohistochemistry on bones sections shows a decrease in the amount of B-ALL cells (brown cells) in mice treated with NVP-BEP800. Counterstaining with Giemsa. Data is representative of four mice, magnification ×20, black scale bars represent 100 µm.

We also used the PDX mice that we developed to investigate to what extent NVP-BEP800 efficiently interfered with B-ALL progression. The first group of mice was injected i.v. with 10 mg/kg of NVP-BEP800 at day 15, 20, and 25 after the transplantation of 100,000 B-ALL cells, while the second group of mice was injected with the vehicle. PDX mice treated with NVP-BEP800 survived longer than PDX mice treated with the vehicle (*P* < 0.001, Fig. 8a). When B-ALL progression was followed in PB at day 35 post-transplantation, we observed a reduced amount of leukemic cells (hCD45^+^ hCD19^+^) in mice treated with NVP-BEP800 (*P* < 0.0001, Fig. 8b). We also generated a mouse model to study bioluminescence. After transplanting bioluminescent B-ALL cells into mice, we observed at day 35 a reduced bioluminescence in the group of mice treated with NVP-BEP800, as well as for bones and spleens (Fig. 8c). Flow cytometry of GFP^+^ B-ALL cells confirmed the reduced proliferation of leukemic cells in the BM of mice treated with NVP-BEP800 on day 35 post-transplantation (*P* < 0.001, Fig. 8d). This effect was confirmed by immunochemistry on BM sections, after hCD19 staining to detect B-ALL cells (Fig. 8e).

In the end, it was found that NVP-BEP800 was effective in vivo, and that treatment of PDX mice delayed the development of T-cell and B-cell ALL.

## Discussion

Within the cell, HSP90 plays a critical role in the proper folding, assembly, and maintenance of the three-dimensional structures of a variety of proteins, referred to as clients. The molecular chaperone HSP90 is a key member of the cellular proteostasis network, and thus helps protect cells from proteotoxic stress. Cancer cells have up-regulated members of this network, including HSP90, to promote their survival and growth. HSP90 inhibition has been shown to be effective in treating lymphomas^15–17^, AML^18–21^, CML ^26–28^, and myeloproliferative neoplasms^24^. Regarding ALL, an interesting study showed that the NOTCH1 signaling status correlated with epichaperome levels and predicted T-ALL cells' response to HSP90 inhibition with the PU-H71 inhibitor^29^. Another study described that NVP-AUY922 led to a degradation of tyrosine kinase 2 (TYK2) signaling and T-ALL apoptosis^30^. In a subset of B-ALL, genetic resistance to JAK2 inhibition was overcome by HSP90 inhibition^31^.

In cancers, epichaperone re-wiring altered a plethora of post-translational interactions and many of which converge upon MYC^59^. However, in our PDX models, T-ALL and B-ALL cells did not express MYC (Supplementary Fig. S15a). In several other cancers, HSP90 inhibitors allowed to discover other client proteins of HSP90, such as STAT3 or AKT^11,52^. These client proteins were not involved in the growth and development of leukemic cells in our PDX models, and neither STAT3 nor AKT were phosphorylated in T-ALL and B-ALL cells (Supplementary Fig. S15a, b). Moreover, drugs that inhibit AKT or STAT3, such as MK-2206 or Niclosamide, did not affect the viability of T-ALL and B-ALL cells (Supplementary Fig. S15c).

Several HSP90 inhibitors have undergone clinical trials, but these drugs, which bound to a shared nucleotide pocket in the N-terminal domain, did not differentiate between four different HSP90 family members: HSP90α, HSP90β, GRP94 (Glucose-regulated protein 94 kDa), and TRAP1 (Tumor necrosis receptor-associated protein 1). Therefore, there was a need to identify chemical compounds that were more specific to HSP90β^60^. NVP-BEP800 was found as a potent inhibitor of HSP90β that was 70-fold less effective against other HSP proteins and many kinases^61^. NVP-BEP800 caused HSP90 dissociation, client proteins degradation and led to growth inhibition or induction of cell death in cancer cell lines^61^. In our study, we discovered that HSP90 was an important regulator of SRC kinases, which were involved in the intracellular signaling pathways necessary for the growth and proliferation of T-ALL and B-ALL cells. Lymphocyte-specific SRC family kinases (SFK) were highly important for both T cells^34–37^ and B cells^39,40^ proliferation. The inhibition of SRC kinases’ phosphorylation, mainly with the use of Bosutinib, Dasatinib, or Saracatinib, was therefore an important strategy for the treatment of T-ALL^36–38^ and B-ALL^41–44^. Using an inhibitor of the chaperone that controls the overall level of SRC is likely a good strategy for the development of therapies based on the SRC kinase inhibition in ALL. In our study, we showed that NVP-BEP800 affected phosphorylated SRC and, simultaneously, the total amount of SRC kinase in cells. It seems however that the total LCK protein was more affected than the total LYN protein, this was observed when both cell lines and ALL cells xenografted in NSG mice were treated with NVP-BEP800. We can therefore assume that HSP90 may interact more with the phosphorylated LYN. Previous studies on AML or myeloproliferative neoplasm cells described the efficiency of co-treatments of HSP90 inhibitors with tyrosine kinase inhibitors^22,25^. In our study, we however observed that co-treatment with Dasatinib, a specific inhibitor of SRC phosphorylation, did not increase in vitro the effect mediated by NVP-BEP800 on the viability of cell lines, as well as on T-ALL or B-ALL cells isolated ex vivo from PDX mice (Supplementary Fig. S16). This is probably because NVP-BEP800, by itself, showed a pertinent dysregulation of phosphorylated SFK.

Most studies with NVP-BEP800 were performed in vitro. In these experiments, a decrease in the migration and invasion of lung carcinoma and glioblastoma cells was observed^62^, along with apoptosis of myeloma cells cultured on stromal support cells^63^, and a reduced proliferation of other tumor cell lines^64^. HSP90 inhibitors, including NVP-BEP800, increased the sensitivity of tumor cells to ionizing radiation^64–66^. In hepatocellular carcinoma, this compound has been found to suppress the vasculogenic networks, which play an important role in tumor malignancy^67,68^. NVP-BEP800 affected the proteasomal degradation of viral HSP90 client proteins, including those required for latency and infectivity of Kaposi sarcoma-associated herpes virus^69^. NVP-BEP800 induced robust antitumor responses on a preclinical xenograft mice model of human breast cancer^61^. Our study showed that NVP-BEP800 has effectively targeted SRC kinases, which should now be considered as novel clients of the HSP90 chaperone. In addition, mice that were treated with NVP-BEP800 survived longer and showed fewer symptoms of leukemia in vivo, confirming that this treatment was effective on both PDX models of T-ALL and B-ALL.

More recent understanding has highlighted that vulnerability of cancer cells to HSP90 inhibitors depends upon pathologic hyperconnectivity within the “epichaperome”, composed of chaperone and co-chaperone complexes, this has been characterized for solid cancers^59,70^, as well as for T-ALL^29^. In our study, we discovered for the first time that HSP90 can bind and control the stability of SRC kinases in ALL, therefore SFK should be considered as important client proteins involved in the epichaperome for T-ALL and B-ALL. Furthermore, lack of predictive biomarkers of HSP90 inhibitors for selecting patients who would show efficacy versus lack of response remains to be characterized for ALL. In our study, we discovered that patient samples showing high levels of phosphorylated SRC were more sensitive in vitro to the HSP90 inhibitor NVP-BEP800, and this might help to predict the response of ALL to HSP90 inhibition.

While NVP-AUY922 and 17-AAG targeted both HSP90α and HSP90β^49,50^, NVP-BEP800 specifically inhibited HSP90β, blocking its N-terminal ATP-binding pocket^48^. Consequently, HSP90 inhibitors that target more precisely HSP90β may have a distinct feature that would favor their clinical development over other HSP90 inhibitors. Previous in vivo studies showed that NVP-BEP800 provided a high degree of flexibility in dose and schedule within the clinical setting, whereas mice in in vivo experiments began to lose body weight when NVP-BEP800 was administered at a dose of greater than 40 mg/kg daily over two weeks^61^. In our experiments, we confirmed the low toxicity of this drug in vivo, by administering three i.v. injections of NVP-BEP800 of 10 mg/kg every five days, and we showed that the drug had no negative effect neither on the development of mice’s body weight (Supplementary Fig. S17a) nor on PB hematopoietic parameters (Supplementary Fig. S17b), as well as on mice’s viability after monitoring the mice for two months.

Constitutive activation of the SFK has been described as important for the proliferation of cancer cells in AML^71–73^. In the present study, in addition to previous descriptions in T-ALL or B-ALL^36–38,41–44^, SRC kinases were also found to be phosphorylated, which attested to the constitutive activation of the SRC kinases for ALL cells, and their importance for the proliferation and growth of ALL cells, in vitro as well as in vivo. An interaction study conducted on HEK293 cells showed that kinases represented the main clients of HSP90, among them the LCK was found^46^. In another work, affinity enrichment of a library of full-length open reading frames allowed to identify LYN kinase interacting partners, among which HSP90 was identified^47^. A TCR-linked multiprotein complex containing HSP90 and LCK has been already described in T-cells^34^. The HSP90-specific inhibitor 17-AAG selectively disrupted kinase-mediated signaling events, including LCK, in normal T-lymphocyte activation^74^. HSP90 has been shown to play a protective role in the regulation of SRC family proteins, as in neutrophils increasing cell survival^75^, or in endothelial cells allowing regulation of the vascular endothelial growth factor receptor^68^. HSP90 bound also to LYN in B-chronic lymphocytic leukemia^45^. In our study, through pull-down assays and treatment with an HSP90 inhibitor, we proved that the SRC kinases LCK and LYN were both clients of HSP90, in T-ALL and B-ALL cells, respectively. We found that the sensitivity of ALL cell lines to NVP-BEP800 was dependent on their expression level of SRC rather than HSP90, and all cell lines expressed HSP90 but only the ones expressing SRC were sensitive to the drug. This was confirmed with the use of lentiviral shRNA tools, as the cells lost their sensitivity to NVP-BEP800 when the expression of LCK or LYN was abolished. This was confirmed for both T-ALL and B-ALL primary cells, as a correlation was observed between their sensitivity to NVP-BEP800 and their expression levels of SRC. Remarkably, in our experiments on cell lines and primary samples, no correlation was observed between HSP90 and SRC protein expression levels. In our study, through the use of NVP-BEP800 and since ALL cells expressing high levels of SFK were more sensitive to HSP90 inhibition, we confirmed that SRC kinases were important to maintain the viability of ALL cells.

Altogether, these findings demonstrated that the chaperoning of SRC kinase by HSP90 contributed to the proliferation and growth of T-ALL and B-ALL cells, which provides novel targeting strategies for ALL treatment. Our promising preclinical test results should be further explored, paving the way for future clinical trials.

## Supplementary information

Related Manuscript File

Supplementary Figures
