# Supplementary material for: HSP90 inhibitor NVP-BEP800 affects stability of SRC kinases and growth of T-cell and B-cell acute lymphoblastic leukemias
Source: Blood Cancer J. 2021 Mar 18;11(3):61. doi: 10.1038/s41408-021-00450-2 (PMC7973815; doi:10.1038/s41408-021-00450-2)
Supplement: Supplementary file 1 — Related Manuscript File [file 41408_2021_450_MOESM1_ESM.docx]

**HSP90 inhibitor NVP-BEP800 affects stability of SRC kinases and growth of T-cell and B-cell acute lymphoblastic leukemia**

Rony Mshaik,^1,2^ John Simonet,^1^ Aleksandra Georgievski,^1^ Layla Jamal,^1^ Shaliha Bechoua,^3^ Paola Ballerini,^4^ Pierre-Simon Bellaye,^5^ Zandile Mlamla,^1,6^ Jean-Paul Pais de Barros,^1,2,6^ Audrey Geissler,^7^ Pierre-Jean Francin,^8^ François Girodon,^1,9^ Carmen Garrido^1,2^ and Ronan Quéré.^1,2,*^

**Supplementary Materials and Methods**

**Cell culture and treatment with NVP-BEP800**

The study was conducted on two T lymphoblast cell lines; Jurkat (Clone E6-1, TIB-152, ATCC) and Rpmi-8402 (CRL-1994, ATCC) as well as four B lymphoblast cell lines; BALL-1 (ACC742, DSMZ), Raji (CCL-86, ATCC), Reh (CRL-8286, ATCC) and Daudi (CCL-213, ATCC). All cell lines were cultured in RPMI-1640 media (Dominique Dutscher) supplemented with 10% fetal bovine serum (Dominique Dutscher) and Penicillin-Streptomycin-Amphotericin (PSA, Pan Biotech). Primary T-ALL and B-ALL cells, preserved in diméthylsulfoxyde (DMSO), were frozen in liquid nitrogen, and then cultured in StemMACS media (Miltenyi Biotec) supplemented with PSA. The same was done for T-ALL and B-ALL cells isolated *ex vivo* from the BM of PDX mice. We always used cells freshly isolated *ex vivo* from the BM in our experiments. ALL cells were also co-cultured with MS5 murine stromal feeder cells (ACC-441, DSMZ), in StemMACS media (Miltenyi Biotec) supplemented with PSA. After treatment, cells were trypsinized and the viability of ALL cells was assessed by Hoechst staining and measured by flow cytometry. The hCD45-APC antibody was used to distinguish between human ALL and mouse MS5 support cells. Cells were grown in an incubator at 37°C in a humid atmosphere and 5% CO_2_ pressure. For viability, cells were treated for 48 hours with increasing concentrations or a single dose of 1μM with NVP-BEP800 (SelleckChem). For cell cycle activity, apoptosis, cell signaling and transcriptional expression experiments, cells were treated for 18 hours with NVP-BEP800. Ethanol is the diluent, which served as a control “vehicle”. T-ALL cells were treated with anti-CD3 (1μg/ml, BE0001-2, BioXcell) and anti-CD28 antibodies (1μg/mL, BE0291, BioXcell) for TCR activation and B-ALL cells were treated with anti-CD40 (1μg/mL, BE0189, BioXcell) for BCR activation prior to NVP-BEP800 treatments. For viability, cells were treated for 48 hours with NVP-BEP800 or Dasatinib (SelleckChem) alone, or with a co-treatment of both compounds.

**Cell viability assay (XTT)**

Viability and proliferation of cell lines were determined using the XTT Cell Viability assay (15960972, CyQUANT XTT Cell Viability Assay, Invitrogen) according to the manufacturer’s instructions. Briefly, cells were seeded in a 96‐well culture plate at a density of 2×10^5^ viable cells (trypan blue negative) in 200µL of media and were treated with increasing concentrations of NVP-BEP800. The culture plates were incubated for 48 hours at 37°C with a 5% CO_2_ atmosphere. After incubation, cells were collected from all the wells and washed with PBS1× (pH 7.4). A mix containing 60µL “XTT Reagent” and 10µL of “Electron Coupling Reagent” was added to each well and the cell plate was incubated for 4 hours. The absorbance was measured at OD 450nm and 660nm with UV-visible spectrophotometer (Biochrom Asys UVM340) using the media as the blank, and the results were determined by the difference in the absorbance values measured at wavelengths of 450nm and 660nm, respectively.

**Western blot**

Cell pellets were suspended in RIPA lysis buffer (150mM NaCl, 5mM EDTA (pH 8.0), 50mM Tris (pH 8.0), 1% NP-40, 0.5% sodium deoxycholate, and 0.1% SDS). On cell lysates, OD 620nm was measured to normalize the amount of the loaded sample. An appropriate quantity of protein was supplemented with 5× Laemmli buffer. Targeted proteins were separated on 10% SDS-PAGE gels and transferred to PVDF membranes. Immunoblot was performed with the indicated antibodies, anti-p-NFϰB(S536) (1:1000, #3033, Cell Signaling Technology), anti-NFϰB (1:1000, #8242, Cell Signaling Technology), anti-p-LYN(Y396) (1:1000, ab226778, Abcam), anti-LYN (1:1000, #2796, Cell Signaling Technology), anti-p-BLK(Y389) (1:1000, PA5-105866, Thermo Fisher Scientific), anti-BLK (1:1000, #3262, Cell Signaling Technology), anti-p-SRC(Y416) (1:1000, #6943, Cell Signaling Technology) was used for p-LCK, anti-pan-SRC (1:1000, #2108, Cell Signaling Technology), anti-LCK (1:1000, #2752, Cell Signaling Technology), anti-p-PLCγ2(Y1217) (1:1000, #3871, Cell Signaling Technology), anti-PLCγ2 (1:1000, #3872, Cell Signaling Technology), anti-p-NFAT1(S54) (1:1000, 44-944G, Thermo Fisher Scientific), anti-NFAT1 (1:1000, 610702, BD Biosciences), anti-HSP90 (1:1000, ADI-SPA-830, Enzo Life Sciences), anti-HSP90α (1:1000, ADI-SPS-771, Enzo Life Sciences), anti-HSP90β (1:1000, ADI-SPA-842, Enzo Life Sciences), anti-HSP70 (1:1000, ADI-SPA-810, Enzo Life Sciences) and anti-ACTB (1:2500, 612656, BD Biosciences). We also used anti-p-STAT3 (Y705) (1:1000, #9145, Cell Signaling Technology), anti-STAT3 (1:1000, #4904, Cell Signaling Technology), anti-p-AKT (S473) (1:1000, #4060, Cell Signaling Technology) and anti-AKT (1:1000, #4691, Cell Signaling Technology), anti-MYC (1:1000, #5605, Cell Signaling Technology), anti-BCL2 (1:1000, Sc-509, Santa Cruz), anti-cleavedCASP3/CASP3 (1:1000, #9665, Cell Signaling Technology). We also used anti-HCK (1:1000, MAB3915, R&D Systems), anti-BTK (1:1000, #8547, Cell Signaling Technology), anti-SYK (1:1000, ab40781, Abcam), anti-ZAP70 (1:1000, #2705, Cell Signaling Technology), anti-JAK3 (1:1000, #8863, Cell Signaling Technology). Appropriate secondary anti-mouse or anti-rabbit antibodies, conjugated with Horseradish Peroxidase were used (1:5000, Cell Signaling Technology). Chemiluminescence was performed (Chemidoc, Bio-Rad), after applying ultra-sensitive enhanced chemiluminescent (ECL) substrate (SuperSignal West Femto Maximum Sensitivity, Thermo Fisher Scientific). Protein sizes were controlled by a protein ladder (Page Ruler Plus Prestained Protein Ladder, Thermo Fisher Scientific), and protein expression levels were assessed by using ImageJ (NIH).

**Immunoprecipitation**

Cell pellets were reconstituted in RIPA lysis buffer. After preclearing with agarose protein A beads (Thermo Fisher Scientific) for 2 hours, cell lysates were incubated with anti-p-SRC(Y416) (1:200, #6943, Cell Signaling Technology) or anti-p-LYN(Y396) (1:200, ab226778, Abcam) antibodies overnight. The antibody/protein complex was then pulled out of the sample using protein A-coupled agarose beads. Agarose beads were washed with the buffer four times, reconstituted in RIPA lysis buffer supplemented with 5× Laemmli buffer, and were then heated at 65°C for 10min. Western blot was performed with the same antibodies and procedure described above, in addition to using secondary anti-rabbit or anti-mouse antibodies specific for immunoprecipitation (TruBlot, Rockland).

**Flow cytometry and fluorescent-activated cell sorting (FACS)**

After tail vein PB sampling from PDX mice, white blood cells were recovered following hemolysis (NH4Cl 150mM, KHCO3 10mM, EDTA 0.1mM, pH 7.4). Bones, tibias and femurs from the two bottom legs were crushed in a mortar and total BM cells were filtered with a sterile cell strainer (70µm). Spleens were also crushed and filtered with the sterile cell strainer in hemolysis solution, and the cells were washed with PBS1×. The development of T-ALL and B-ALL in NSG mice was characterized in PB and BM by flow cytometry using the following anti-human antibodies; anti-hCD45-APC (1:100, 130-110-633, Miltenyi Biotec), anti-CD7-VioBright-FITC (1:100, 130-123-864, Miltenyi Biotec) for T-ALL and anti-CD19-VioBright-515 (1:100, 130-113-175, Miltenyi Biotec) for B-ALL detection. These antibodies were also used to distinguish between human ALL cells and murine MS5 cells *in vitro*. We also used anti-CD28-PE-Vio770 (130-104-189, Miltenyi Biotec) and anti-CD3-PerCP (345766, BD Biosciences) antibodies to analyze the expression of specific markers by T-ALL cells, as well as an anti-CD40 (1:100, BE0189, BioXcell) antibody with secondary anti-mouse-AF488 (1:500, Thermo Fisher Scientific) for B-ALL cells. To study cell cycle and apoptosis, we used anti-Ki67-FITC (1:20, 556026, BD Biosciences), anti-Ki67-BV421 (1:50, 562899, BD Biosciences), 7-AAD (1:20, 559925, BD Biosciences) and anti-Annexin-V-APC (1:50, 550475, BD Biosciences). For intracellular protein staining, anti-HSP90-DyLight-488 (1:200, ADI-SPA-830-488, Enzo Life Sciences) and anti-p-panSRC-AF647 (1:200, 560096, BD Biosciences) were used after permeabilization. On primary ALL cells, we used an anti-p-SRC-AF647 (1:200, 560096, BD Biosciences) antibody after cells’ permeabilization. This antibody detects p-LCK, p-LYN, p-HCK, p-FYN and p-YES1, therefore we performed a TaqMan assay to confirm a specific expression of LCK by T-ALL cells and LYN by B-ALL cells. To show that T-ALL and B-ALL cells in PDX mice expressed high levels of HSP90 and SRC, we also used an anti-SRC (1:200, #2108, Cell Signaling Technology) antibody followed by a secondary anti-rabbit-AF568 (1:500, Thermo Fisher Scientific) antibody. After cell surface staining, cells were fixed and permeabilized using BD Cytofix/Cytoperm Plus Fixation/ Permeabilization Kit (BD Biosciences). Viability was controlled with Hoechst (Life technologies). When cells were permeabilized for intracellular staining, a Fixable Viability Stain 450 was used (FVS450, BD Biosciences). Cell subsets were analyzed using a Canto10 or a LSR-Fortessa (BD Biosciences). Cells were sorted on a FACS Aria cell sorter (BD Biosciences) equipped with BD FACSDiva software (BD Biosciences). Data were analyzed using FlowJo software (V10, TreeStar Inc).

**Fluorescence microscopy**

ALL cells were purified using anti-hCD45 microbeads (130-045-801, Miltenyi Biotec) on an AutoMACS (Miltenyi Biotec). The cells extracted from BM were permeabilized using BD Cytofix/Cytoperm Plus Fixation/ Permeabilization Kit (BD Biosciences) and then stained with anti-HSP90-DyLight-488 (1:200, ADI-SPA-830-488, Enzo Life Sciences) antibody and anti-SRC (1:200, #2108, Cell Signaling Technology) followed by a secondary anti-rabbit-AF568 (1:500, Thermo Fisher Scientific) antibody. In addition, on BM ALL cells, we used anti-NFAT1 (1:500, 610702, BD Biosciences) and anti-NFϰB (1:500, #8242, Cell Signaling Technology) for intracellular staining, followed by a secondary anti-rabbit-AF488 (1:500, Thermo Fisher Scientific) antibody. Hind limb bones were collected, stripped of soft tissue, fixed and decalcified in a specific buffer (#3800400, Decalcifier-I, Leica) for 48 hours, processed and embedded in paraffin. Thick sections were cut from paraffin-embedded samples and used for fluorescent staining with mouse anti-HSP90-DyLight-488 (1:200, ADI-SPA-830-488, Enzo Life Sciences), rabbit anti-hCD7 (1:200, ab109296, Abcam) or rabbit anti-hCD19 (1:200, SAB5500047, Sigma-Aldrich), followed by anti-rabbit AF568 antibody (Life Technologies). ALL cells and bone sections were fixed with ProLong Gold Antifade reagent containing DAPI (P36931, Thermo Fisher Scientific). Images were acquired with an Axio Imager M2 (Zeiss) coupled with an Apotome.2 and processed for studies (Fiji, NIH software).

**Immunohistochemistry**

Hind limb bones were collected, stripped of soft tissue, fixed and decalcified (#3800400, Decalcifier-I, Leica) for 48 hours, processed and embedded in paraffin. Spleens were fixed in 10% buffered formalin for 48 hours and embedded in paraffin. Thick sections were cut from paraffin-embedded samples and used for immunohistochemistry. We used rabbit anti-hCD19 (1:100, SAB5500047, Sigma-Aldrich) or rabbit anti-hCD7 (1:100, ab109296, Abcam) followed by a secondary anti-rabbit antibody, conjugated with Horseradish Peroxidase (#MP-7401, ImmPRESS HRP anti-rabbit IgG polymer detection kit, Vector laboratories) and substrate (#SK-4800, vector, NovaRED substrate kit, Vector laboratories).

**Quantitative reverse transcription PCR**

After mRNA isolation with the RNeasy kit (Qiagen) or mRNA organic extraction with Qiazol (Qiagen), Moloney murine leukemia virus reverse transcriptase (M-MLV RT, Promega) was used to synthesize cDNA. The following TaqMan assays were then used for TaqMan qPCR: BCL2 (Hs00608023), BIM (Hs00708019), BCL2L1 (Hs00236329), BAD (Hs00188930), BAX (Hs00180269), CDKN1A (Hs00355782_m1), CDKN1B (Hs00153277), PIM1 (Hs01065498), PIM2 (Hs00179139), CCND2 (Hs00153380), CCND3 (Hs01017690), c-MYC (Hs00153408), LCK (Hs00178427), LYN (Hs01015819), HCK (Hs01067403), FYN (Hs00941613), YES1 (Hs01080050) and ACTB (Hs01060665) used as an endogenous control. We used TaqMan Gene Expression Master Mix (Applied Biosystems). Experiments were carried out using the Viia7 system (Applied Biosystems).

**shRNA lentiviral cloning, production and transduction**

Control lentiviral vector (#111170, Addgene) contained shRNA-targeting Renilla luciferase (Ren.713). Lentiviral vectors carrying shLCK or shLYN targeting LCK or LYN genes were generated, following a previously described protocol,^1^ by cloning a 97bp shRNA template sequences for LCK; TGCTGTTGACAGTGAGCGAAGGCATCAAGTTGACCATCAATAGTGAAGCCACAGATGTATTGATGGTCAACTTGATGCCTGTGCCTACTGCCTCGGA and for LYN; TGCTGTTGACAGTGAGCGACAGATTTGTTTTGACAATGTATAGTGAAGCCACAGATGTATACATTGTCAAAACAAATCTGGTGCCTACTGCCTCGGA into the GFP-(miR-E)-PGK-Puro vector (#111170, Addgene) after removing the Ren.713. All constructs were verified by sequencing (Genewiz) and lentiviruses were produced in HEK293 cells after transduction with Lipofectamin 2000 (Thermo Fisher Scientific) of the GFP-(miR-E)-PGK-Puro vector, as well as PAX2 (Addgene, #12260) and pCMV-VSV-G (Addgene, #8454) plasmids. After two days, viral supernatants were recovered and six-well plates were incubated 4 hours with retronectin (Takara, Ozyme). Viral supernatants were then spinoculated for 30min at 4,000g. Cells were cultured on these plates for three days in RPMI-1640 media (Dominique Dutscher). Lentiviral transduced cells (GFP^+^) were sorted on a FACSAriaIII cell sorter (BD Biosciences). The knockdown efficiency was determined by western blot.

**High-performance liquid chromatography (HPLC)**

HPLC was first performed on a serial dilution of NVP-BEP800 in PBS1× *in vitro* to establish a correlation between peak areas measured by HPLC and concentration of NVP-BEP800 *in vitro*. Then concentrations in plasma, BM, kidneys and liver were established. One hour after the injection of NVP-BEP800 at 10mg/kg, mice were anesthetized for tail vein bleeding. Plasma was recovered after a centrifugation of PB at 10,000g for 10min. BM was recovered from bones crushed in a mostar, two bottom legs (tibas and femurs) were crushed in 1mL of PBS1×. The volume of BM was estimated at 5µL. Kidneys and liver were crushed in 3 volumes of PBS1×. Following quantification of NVP-BEP800 by HPLC, concentrations in BM, kidneys and liver were calculated, taking into account the dilution in PBS1×. Preparation of standards and extracts; NVP-BEP800 stock solution (5mM in ethanol) was used to prepare eight calibrant standards (0.078 to 10pmol/µL in 50% Acetonitrile). NVP-BEP800 recovery was assessed by mixing 100µL of each standard with 100µL of either PBS, control plasma or control BM samples. Protein precipitation was achieved at -80°C for one hour with 800µL of ethanol. After a centrifugation step (15min at 15,000g, 4°C) supernatants were collected, evaporated to dryness, and suspended in 100µl of 50% Acetonitrile. Solubilized extracts were further centrifuged at 10,000g for 5min, and supernatant were transferred into autosampler vials. For NVP-BEP800 analysis by Ultra-High Performance Liquid Chromatography (UHPLC), we used a DIONEX Ultimate 3000 UHPLC system equipped with a DGP-3600RS pump, a WPS-3000TRS autosampler and a DAD-3000 detector (Thermo-Fisher, USA). NVP-BEP800 standards and samples (10µL) were analyzed using a Poroshell C8 100×2.1mm, 2.7µm column (Agilent Technologies) at a flow rate of 0.6mL/min, 50°C, with a linear gradient of phosphoric acid (0.1%v/v) buffered ultrapure water (solvent A) and acetonitrile (solvent B) as follows: 10% B for 1min, ramped up to 95% in 4min and held at 95% for 3min. Acquisition was performed at 345nm. Instrument control, data acquisition and analyses was performed with Chromeleon version 7.2.9.

1 Fellmann, C. *et al.* An optimized microRNA backbone for effective single-copy RNAi. *Cell Rep* **5**, 1704-1713, doi:10.1016/j.celrep.2013.11.020 (2013).
