# Supplementary material for: HSP90 inhibitor NVP-BEP800 affects stability of SRC kinases and growth of T-cell and B-cell acute lymphoblastic leukemias
Source: Blood Cancer J. 2021 Mar 18;11(3):61. doi: 10.1038/s41408-021-00450-2 (PMC7973815; doi:10.1038/s41408-021-00450-2)
Supplement: Supplementary file 2 — Supplementary Figures [file 41408_2021_450_MOESM2_ESM.docx]

**HSP90 inhibitor NVP-BEP800 affects stability of SRC kinases and growth of T-cell and B-cell acute lymphoblastic leukemia**

Rony Mshaik,^1,2^ John Simonet,^1^ Aleksandra Georgievski,^1^ Layla Jamal,^1^ Shaliha Bechoua,^3^ Paola Ballerini,^4^ Pierre-Simon Bellaye,^5^ Zandile Mlamla,^1,6^ Jean-Paul Pais de Barros,^1,2,6^ Audrey Geissler,^7^ Pierre-Jean Francin,^8^ François Girodon,^1,9^ Carmen Garrido^1,2^ and Ronan Quéré.^1,2,*^

**Supplementary Figures**


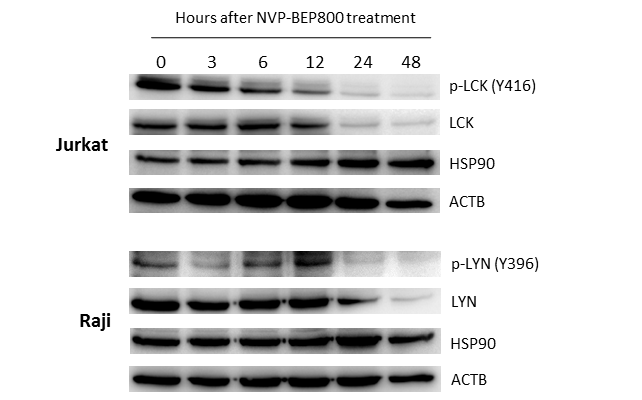


**Supplementary Fig. S1.** In a time course experiment, western blot showing loss of LCK and LYN expression between 12 and 24 hours after treatment with NVP-BEP800 (1μM) on Jurkat and Raji cell lines.


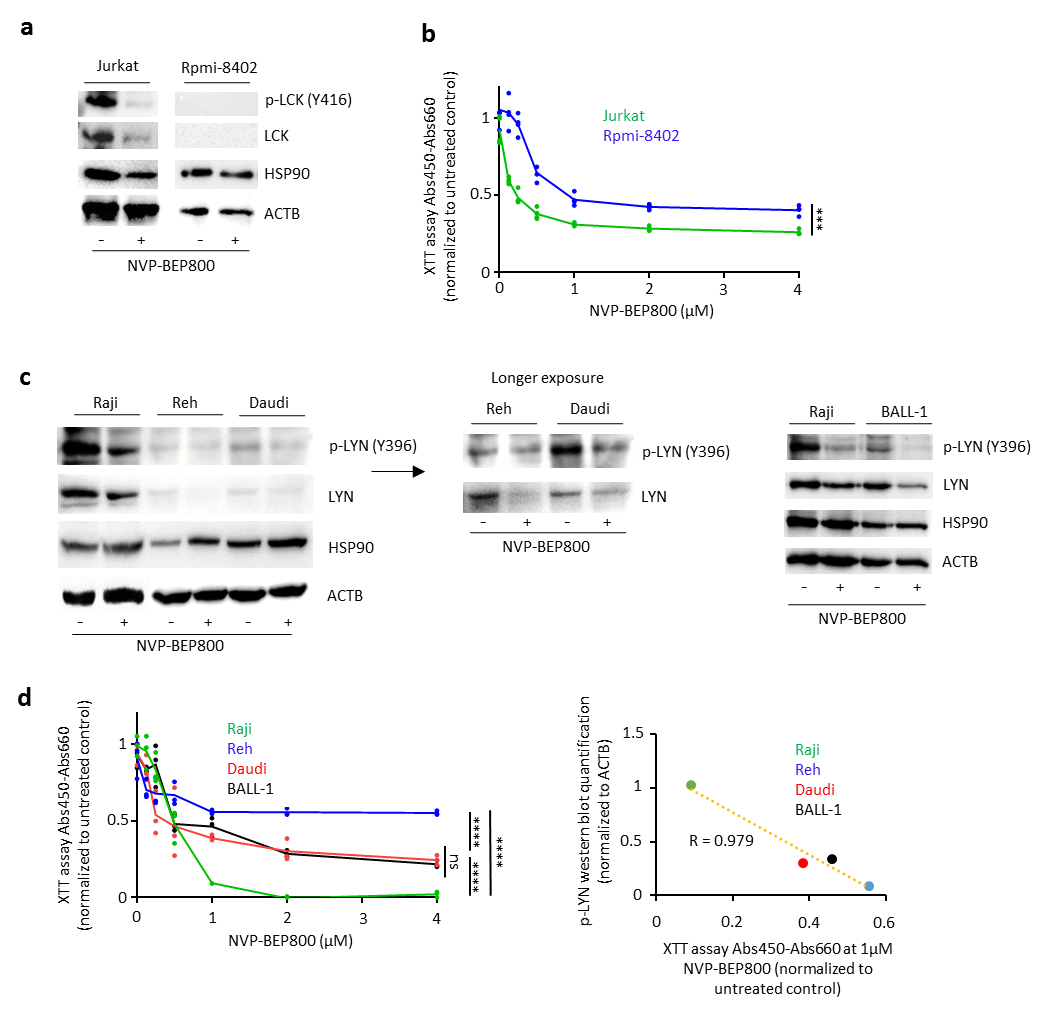


**Supplementary Fig. S2.** Sensitivity of T and B lymphoblast cell lines to NVP-BEP800 is dependent on SRC expression. (**a**) Western blot shows that the Jurkat cells express more LCK, compared to Rpmi-8402 cells. The LCK kinase is affected after treatment with NVP-BEP800 (1µM for 18 hours). (**b**) The XTT viability assay shows that Jurkat cells are more sensitive to NVP-BEP800, compared to Rpmi-8402 cells, after 48 hours of treatment. Data are shown as mean. P value measured by two-tailed unpaired Student’s t-test; ***, P<0.001. (**c**) Western blot shows that out of the four B lymphoblast cell lines, Raji cells expressed high level of p-LYN, while Daudi and BALL-1 cells shows intermediate rates, and Reh cells shows low level. Moreover, cells treated with NVP-BEP800 at 1µM for 18 hours display a reduction in LYN and p-LYN expression. (**d**) XTT viability assay, after 48 hours of treatments, showing that the sensitivity of the four B lymphoblast cell lines to NVP-BEP800 correlates with the expression level of p-LYN. Data are shown as mean (left panel). P value measured by one-way Anova test with Tukey’s multiple comparison test; ****, P<0.0001; ns, non-significant.


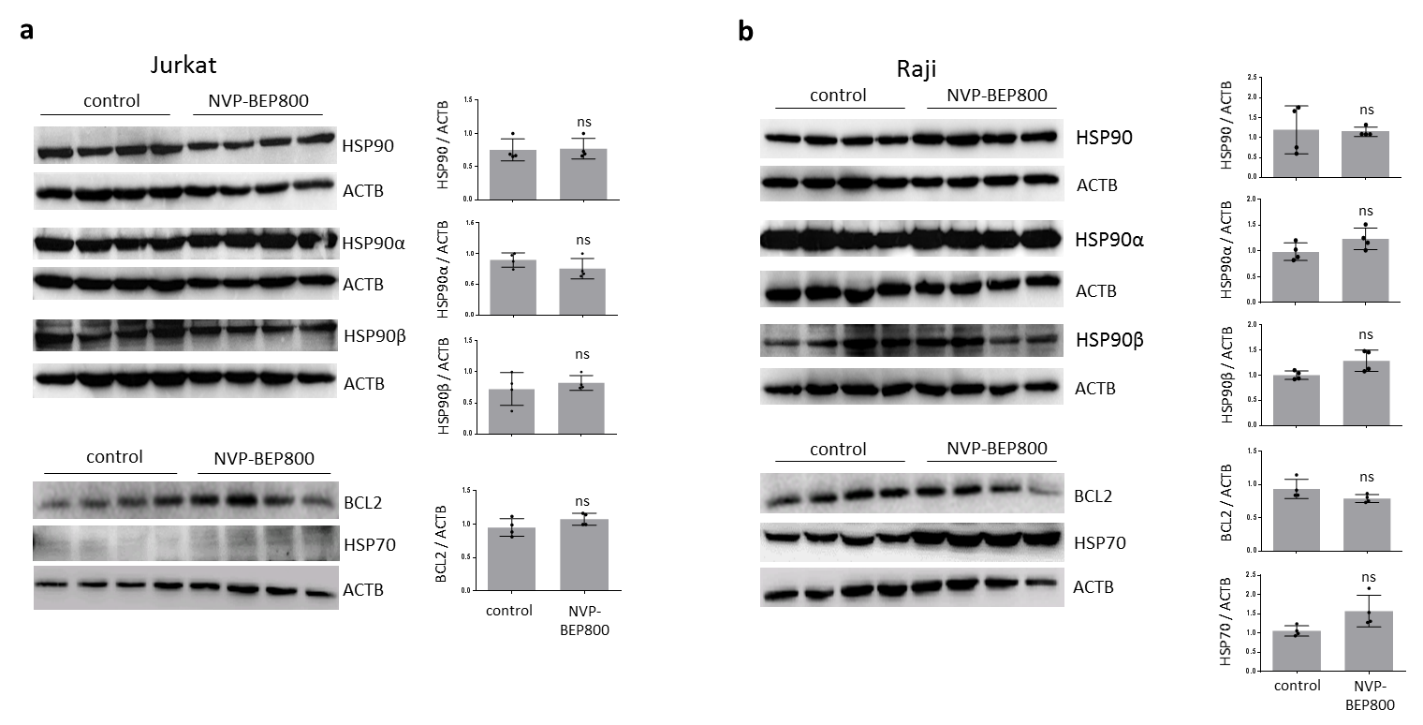


**Supplementary Fig. S3.** NVP-BEP800 does not affect expression of HSP90, HSP90α, HSP90β, HSP70, or BCL2. Western blot shows that NVP-BEP800 (1µM for 18 hours) does not affect expression of these proteins in Jurkat (**a**) and Raji (**b**) cells. The quantification normalized to ACTB is shown on the right panel. Data shows mean ± SD; n=4. P value measured by two-tailed unpaired Student’s t-test; ns, non-significant.

**
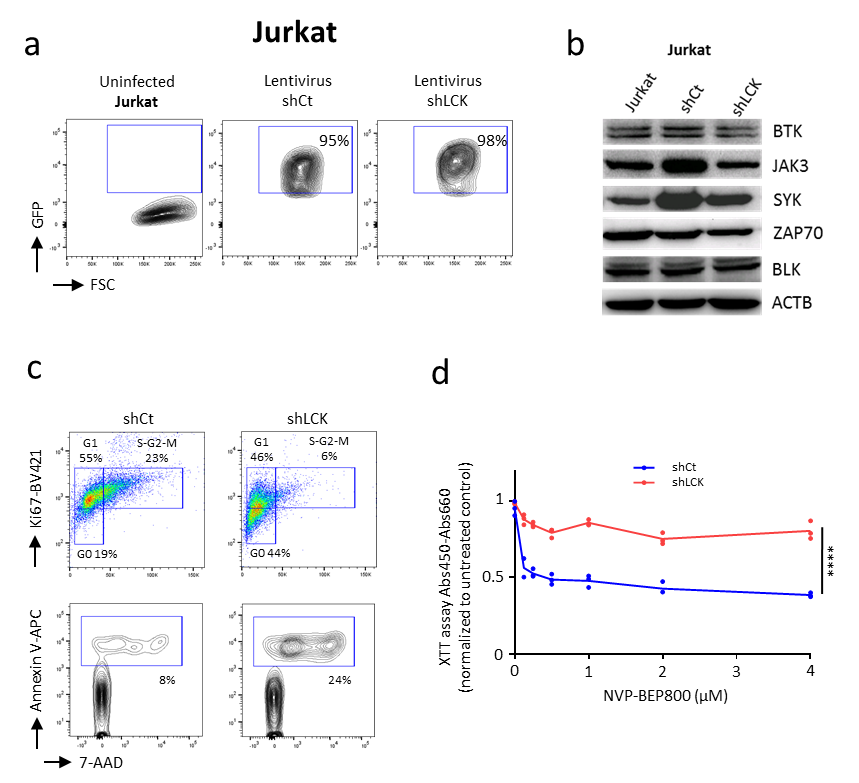
**

**Supplementary Fig. S4.** Down regulation of LCK kinase inhibits NVP-BEP800 efficiency on T-ALL Jurkat cells. (**a**) Flow cytometry on Jurkat cells transduced with the shCt or shLCK through lentiviral infection followed by FACS to select GFP^+^ cells, as shown in Figure 2a,c. (**b**) Specificity of the shRNAs for LCK (shown in Figure 2a), over other tyrosine kinases. (**c**) Analysis of cell cycle and apoptosis showing a reduction in the percentage of Jurkat cells in the active phase of division (Ki67^+^ 7-AAD^+^) and an increase in apoptosis (Annexin V^+^) of Jurkat cells expressing shLCK, maintained for 7 days *in vitro*. (**d**) XTT viability assay showing lower sensitivity of shLCK Jurkat cells to NVP-BEP800. 2×10^5^ viable cells (trypan blue negative) are seeded and the absorbance is measured to determine viability 48 hours after treatment. Data are shown as mean; n=3. P value measured by two-tailed unpaired Student’s t-test; ****, P<0.0001.

**
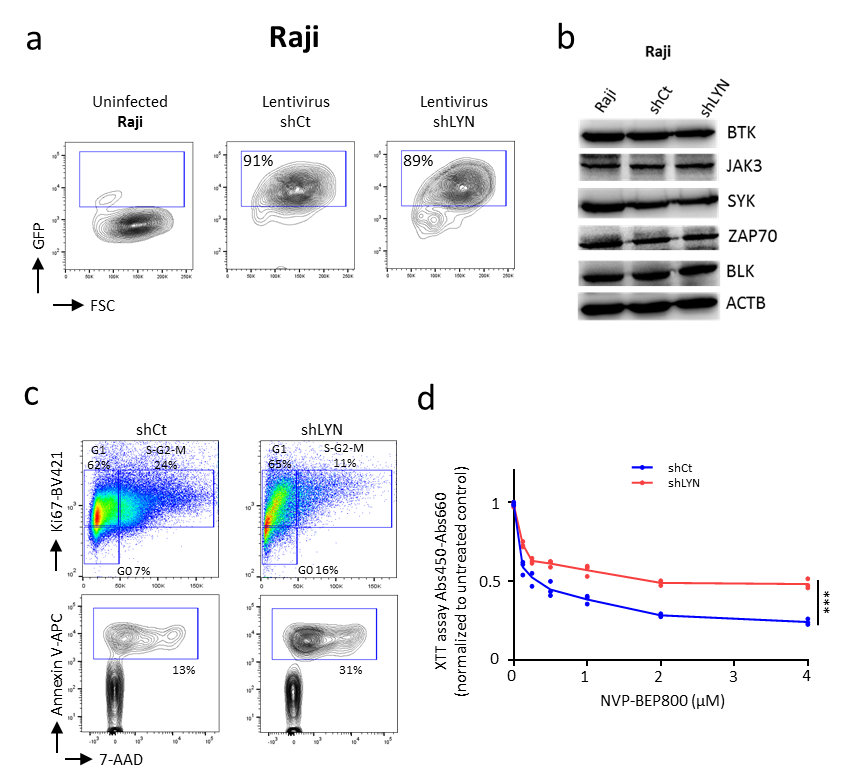
**

**Supplementary Fig. S5.** Down regulation of LYN kinase inhibits NVP-BEP800 efficiency on B-ALL Raji cells. (**a**) Flow cytometry on Raji cells transduced with the shCt or shLYN through lentiviral infection followed by FACS to select GFP^+^ cells, as shown in Figure 2b, d. (**b**) Specificity of the shRNAs for LYN (shown in Figure 2b), over other tyrosine kinases. (**c**) Analysis of cell cycle and apoptosis showing a reduction in the percentage of Raji cells in the active phase of division (Ki67^+^ 7-AAD^+^) and an increase in apoptosis (Annexin V^+^) of Raji cells expressing shLYN, maintained for 7 days *in vitro*. (**d**) XTT viability assay showing lower sensitivity of shLYN Raji cells to NVP-BEP800. 2×10^5^ viable cells (trypan blue negative) are seeded and the absorbance is measured to determine viability 48 hours after treatment. Data are shown as mean; n=3. P value measured by two-tailed unpaired Student’s t-test; ***, P<0.001.


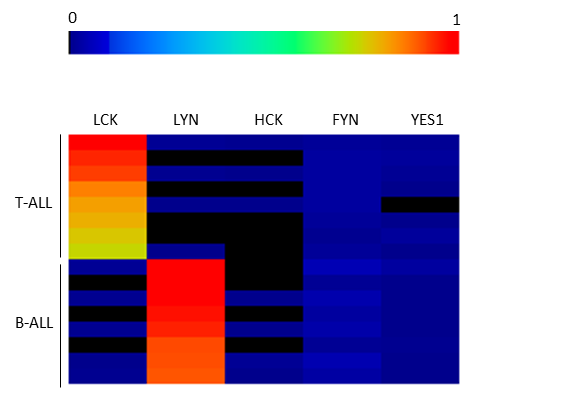


**Supplementary Fig. S6.** RTqPCR shows specific expression of *LCK* by T-ALL primary cells (n=8 samples) and *LYN* expression by primary B-ALL cells (n=8 samples), among further Lymphocyte-specific SRC family kinases (SFK). Samples tested were the same samples presented in Figure 3C.

**Supplementary Fig. S7.** Biological and molecular characteristics of primary T-ALL and B-ALL samples, sorted according to their sensibility to NVP-BEP800 (percentage of viable cells after treatment with NVP-BEP800). CR: cytological remission. (t) translocation, (a) intragenic amplification, (d) deletion, (m) mutation, (f) fusion. BM (bone marrow), PB (peripheral blood). M (male), F (female), cohort of Dijon (D), cohort of Paris (P).


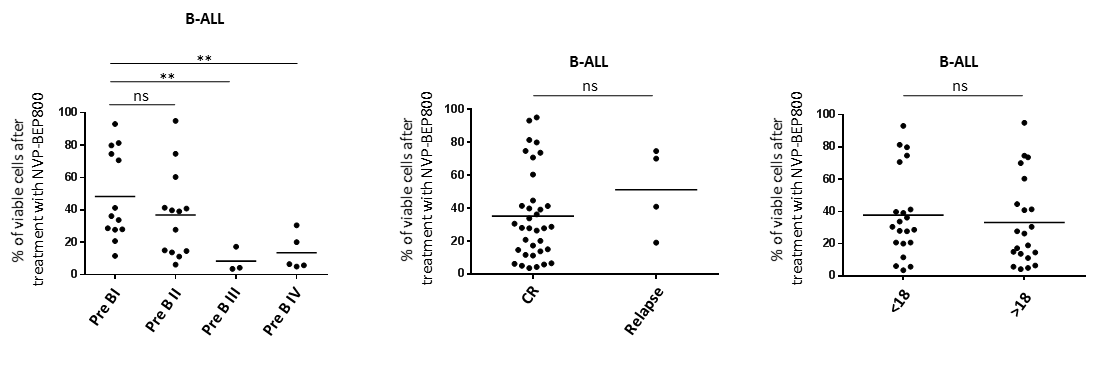


**Supplementary Fig. S8.** A statistic on primary B-ALL samples, showing the percentage of viable cells after treatment with NVP-BEP800 in different groups; immunophenotype, outcome after treatment (CR: cytological remission) or age of the patients (<18 or >18 years old). P value measured by Mann Whitney test; **, P<0.01; ns, non-significant.


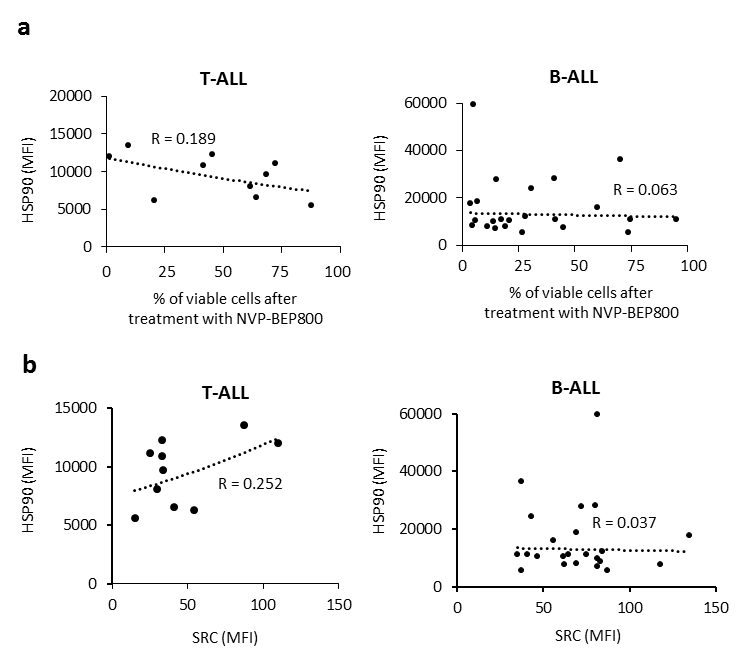


**Supplementary Fig. S9.** (**a**) A scatter plot showing that the percentage of viable cells after treatment with NVP-BEP800 does not correlate with expression levels of HSP90 measured by flow cytometry, for primary T-ALL and B-ALL samples. (**b**) A scatter plot showing that expression measured by flow cytometry for HSP90 and SRC are not correlated, for primary T-ALL and B-ALL samples. Median fluorescence intensity (MFI).

**
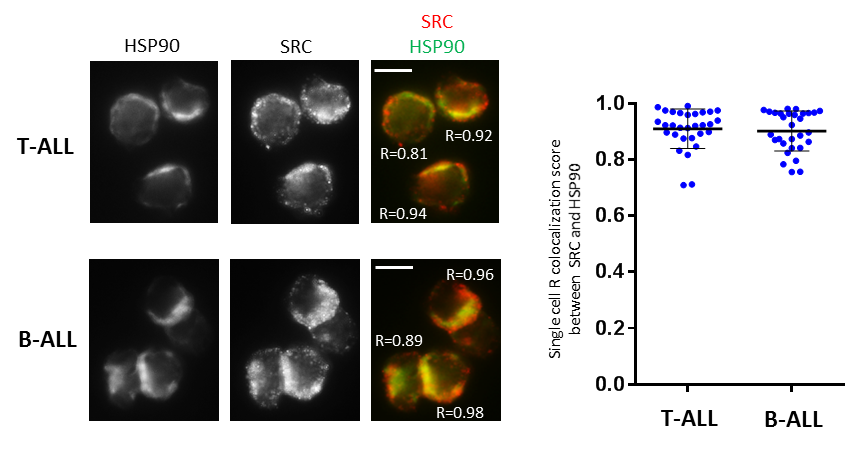
**

**Supplementary Fig. S10.** Microscopy showing colocalization between HSP90 and SRC in the cytoplasm for T-ALL and B-ALL cells extracted *ex vivo* using hCD45 microbeads. Example of three cells observed under the microscope on the left panel (Magnification ×63, white scale bars represent 5µm) and colocalisation score (R) observed for single cells (n=30) on the right panel.

**
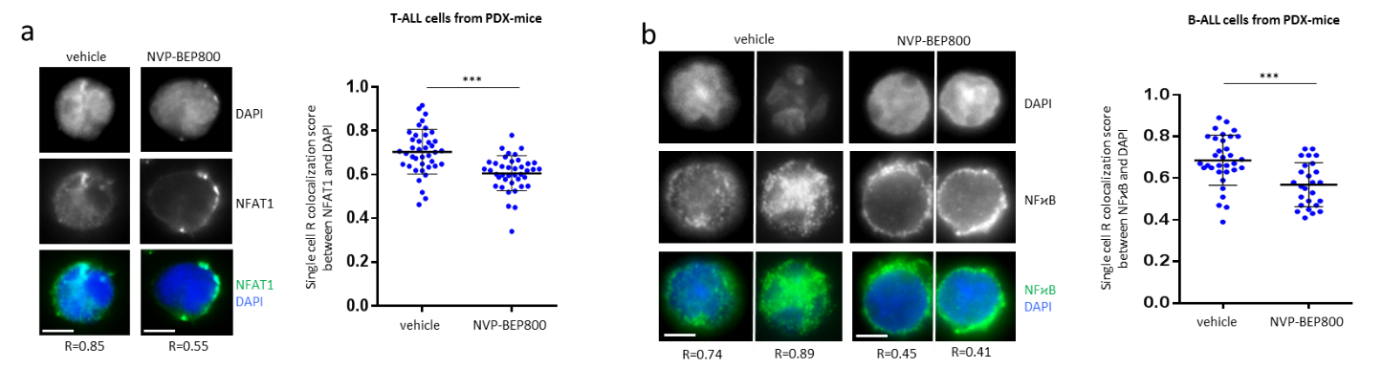
**

**Supplementary Fig. S11.** (**a**) Immunofluorescence imaging showing that NFAT1 is excluded from the nucleus after a treatment with NVP-BEP800 (1µM) on T-ALL cells isolated from PDX-mice. Microscopy showing colocalization between NFAT1 and DAPI (nucleus) on T-ALL cells. Example of single cells observed under the microscope on the left panel (Magnification ×63, white scale bars represent 5µm) and colocalisation score (R) observed for single cells (n>30) on the right panel. Data shows mean ± SD. P value measured by two-tailed unpaired Student’s t-test; ***, P<0.001. (**b**) Immunofluorescence imaging showing that NFϰB is excluded from the nucleus after a treatment with NVP-BEP800 (1µM) on B-ALL cells isolated from PDX-mice. Microscopy showing colocalization between NFϰB and DAPI (nucleus) on B-ALL cells. Example of single cells observed under the microscope on the left panel (Magnification ×63, white scale bars represent 5µm) and colocalisation score (R) observed for single cells (n>30) on the right panel. Data shows mean ± SD. P value measured by two-tailed unpaired Student’s t-test; ***, P<0.001.


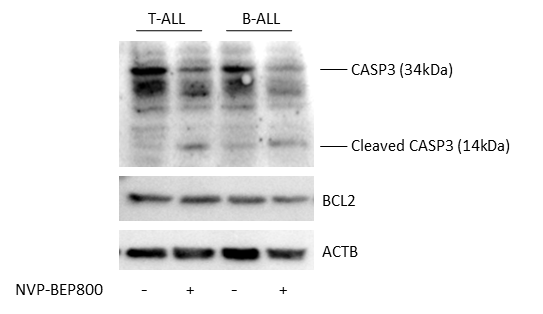


**Supplementary Fig. S12.** NVP-BEP800 induces apoptosis of T-ALL and B-ALL cells as assessed by western blot showing cleaved Caspase 3 (CASP3) after treatment with NVP-BEP800 (1µM for 18 hours).

**
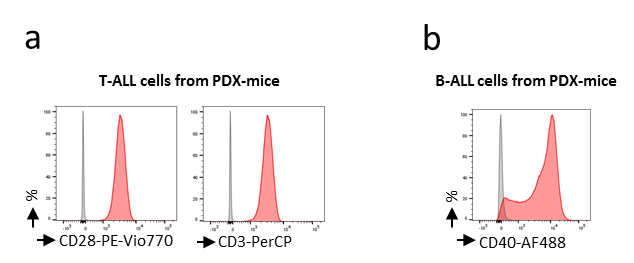
**

**Supplementary Fig. S13.** (**a**) Flow cytometry of T-ALL cells recovered from PDX-mice showing expression of CD3 and CD28. (**b**) Flow cytometry of B-ALL cells recovered from PDX-mice showing expression of CD40.


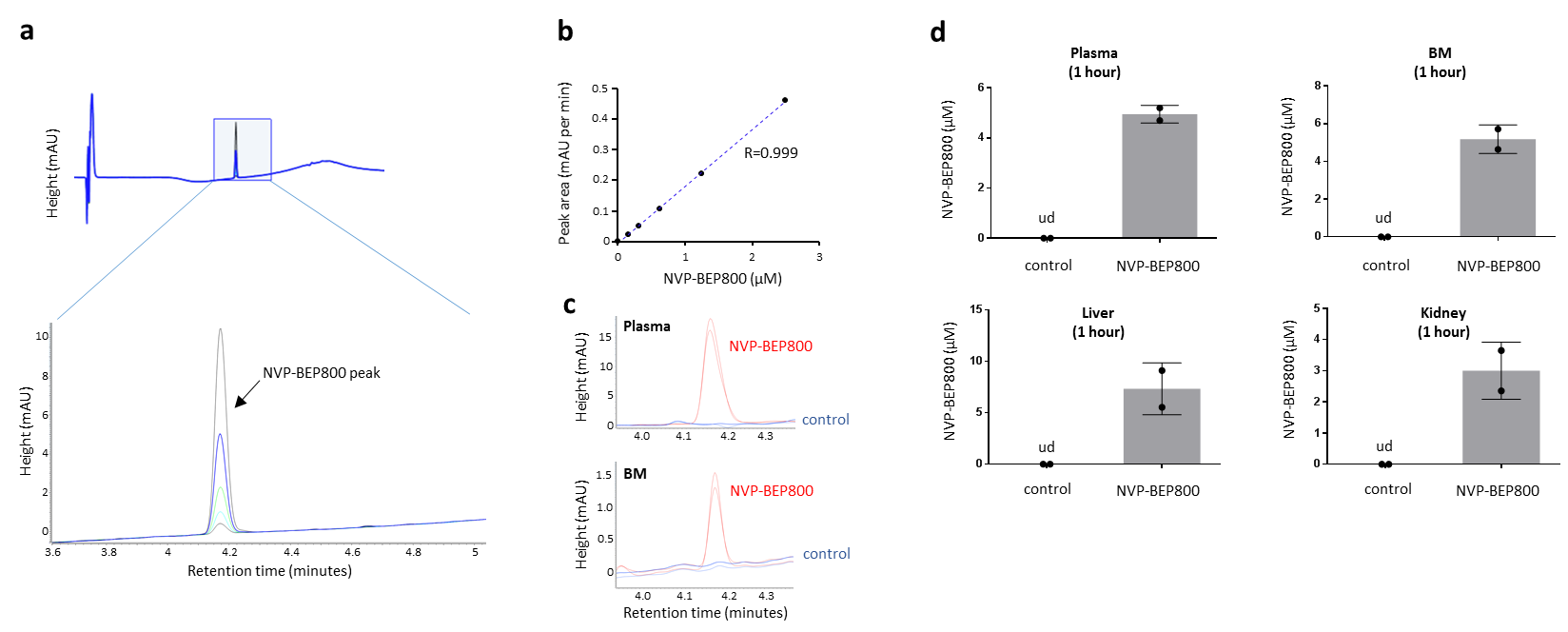


**Supplementary Fig. S14.** Pharmacokinetic study of NVP-BEP800 *in vivo* by high-performance liquid chromatography (HPLC). (**a**) HPLC results of the serial dilution of NVP-BEP800 in PBS1× *in vitro*. (**b**) Correlation between peak areas measured by HPLC and concentration of NVP-BEP800 *in vitro*. (**c**) Result of the HPLC on plasma and BM samples *in vivo*, mice were analyzed one hour after NVP-BEP800 injection (i.v.) at 10mg/kg. Data shows results from two control mice (blue lines) and two mice injected with NVP-BEP800 (red lines). (**d**) Quantification of NVP-BEP800 *in vivo*, in different tissues; plasma, BM, liver and kidney, one hour after the injection. ud, undetected. Data shows mean ± SD.


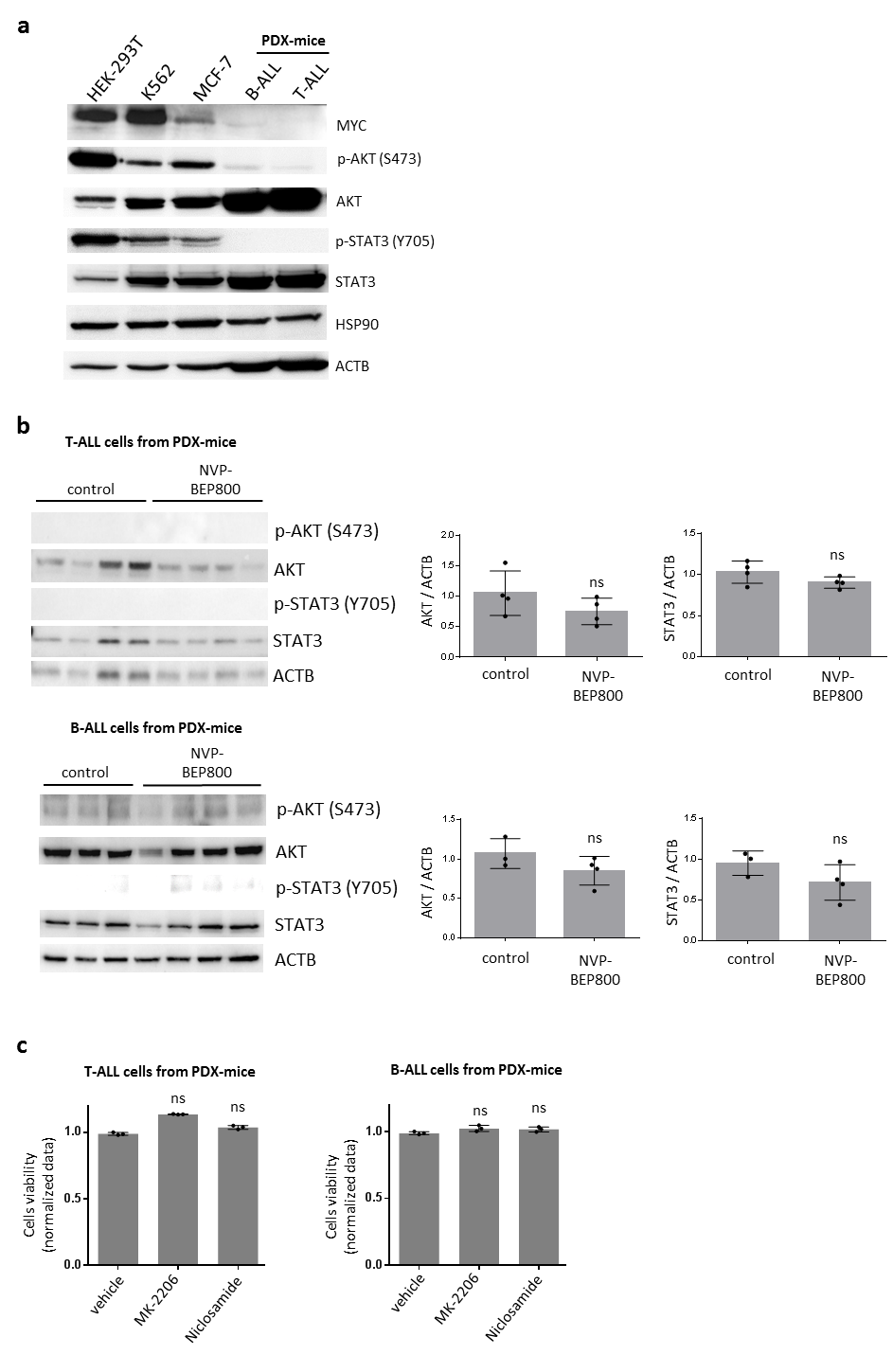


**Supplementary Fig. S15.** T-ALL and B-ALL cells recovered from PDX-mice show no expression of MYC, as well as no activation of STAT3 and AKT pathways. (**a**) Western blot showing that T-ALL and B-ALL cells do not express MYC, as well as phosphorylated STAT3 (Y705) and AKT (S473). Three cell lines were used as positive controls. (**b**) Treatment with NVP-BEP800 does not affect the stability of AKT and STAT3 proteins. The quantification of AKT and STAT3 normalized to ACTB is shown on the right panel. Data shows mean ± SD; n=3-4. P value measured by two-tailed unpaired Student’s t-test; ns, non-significant. (**c**) The viability of T-ALL and B-ALL cells recovered from PDX-mice remains unchanged after *in vitro* treatment with inhibitors of AKT (MK-2206, 1µM) or STAT3 (Niclosamide, 1µM). Viability is assessed by flow cytometry. Data shows mean ± SD; n=3. P value measured by one-way Anova test with Tukey’s multiple comparison test; ns, non-significant.


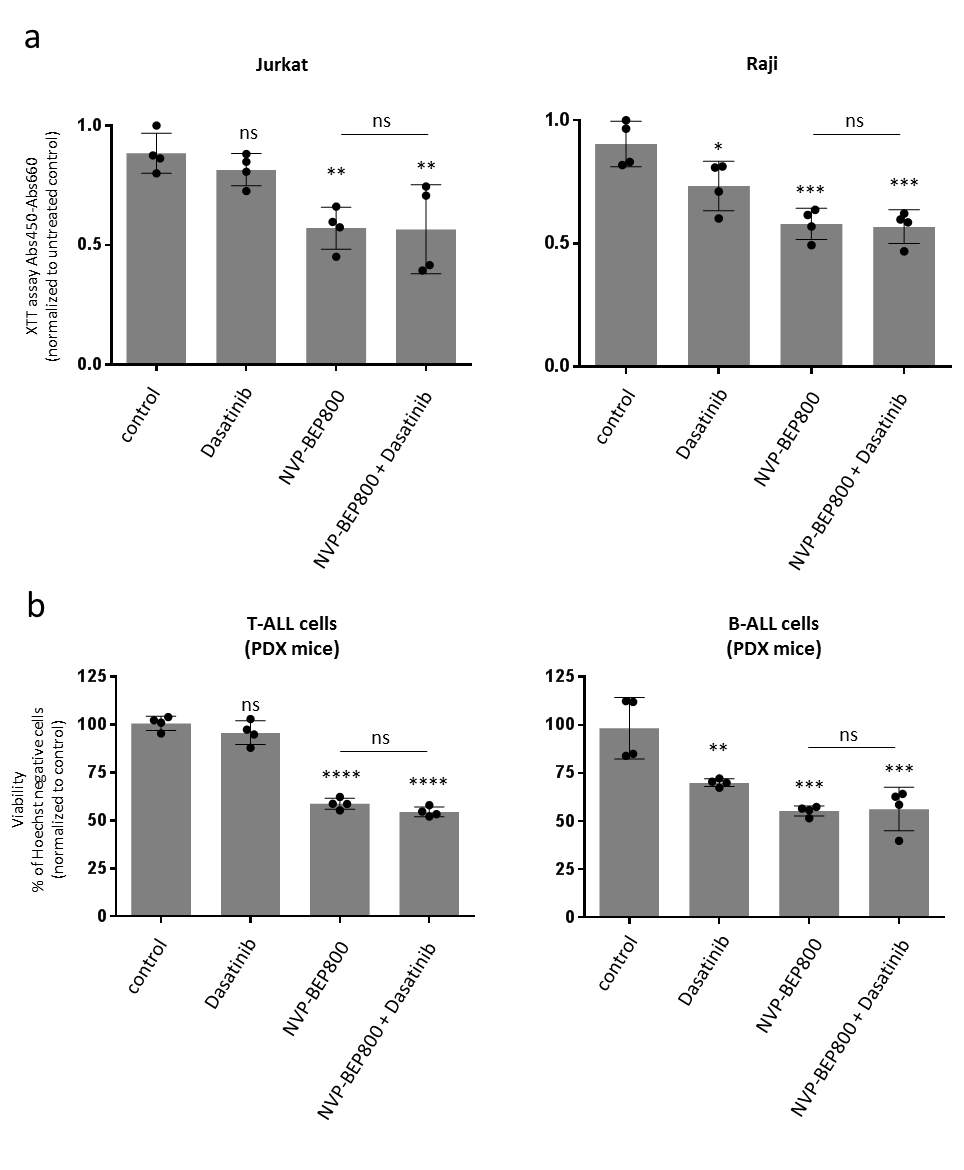


**Supplementary Fig. S16.** XTT viability assay showing that a co-treatment of cells with Dasatinib, a specific inhibitor of SRC phosphorylation, and NVP-BEP800 did not increase the *in vitro* effect mediated by this latter alone. (**a**) Data obtained for Jurkat and Raji cells. 2×10^5^ viable cells are seeded and the absorbance is measured to determine viability 48 hours after treatment with Dasatinib (1µM), NVP-BEP800 (1µM) or a co-treatment (1µM each). Data are shown as mean ± SD; n=4. P value measured by one-way Anova test with Tukey’s multiple comparison test; *, P<0.05; **, P<0.01; ***, P<0.001; ns, non-significant. (**b**) Data obtained for T-ALL and B-ALL cells, isolated *ex vivo* from the BM of PDX mice. 10^6^ cells are seeded and the viability is determined by flow cytometry, 48 hours after treatment with Dasatinib (1µM), NVP-BEP800 (1µM) or a co-treatment (1µM each). Data are shown as mean ± SD; n=4. P value measured by one-way Anova test with Tukey’s multiple comparison test; **, P<0.01; ***, P<0.001; ***, P<0.0001; ns, non-significant.


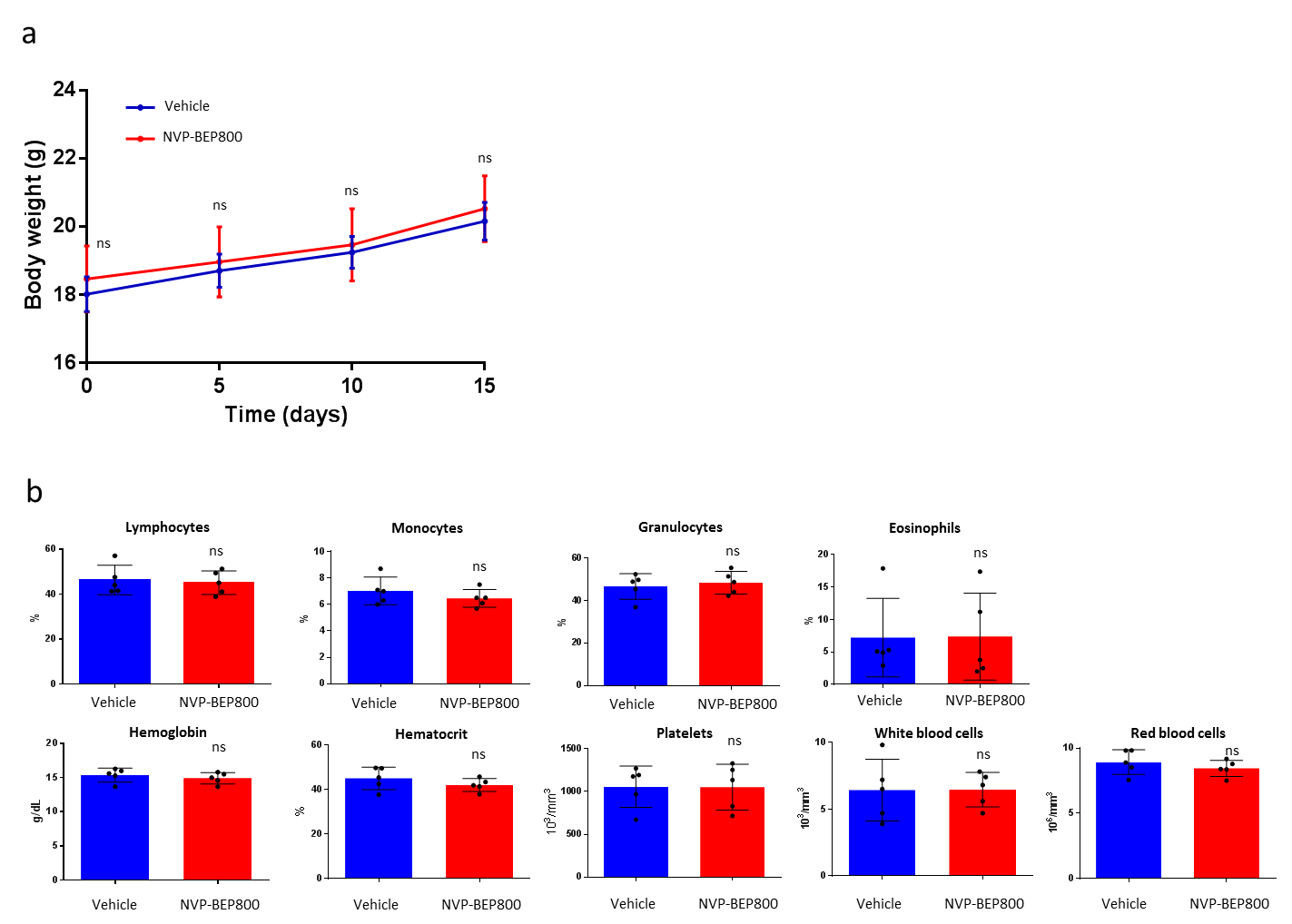


**Supplementary Fig. S17.** NVP-BEP800 presents no toxicity on NSG mice. (**a**) NVP-BEP800 has no effect on the development of mice's body weight after three i.v. injections of the drug at a dose of 10mg/kg at days 0, 5 and 10. Data shows mean ± SD; n=5. P value measured by two-tailed unpaired Student’s t-test; ns, non-significant. (**b**) NVP-BEP800 has no effect on PB parameters, when PB is analyzed at day 15, by using a hemocytometer. Data shows mean ± SD; n=5. P value measured by two-tailed unpaired Student’s t-test; ns, non-significant.
